# Transcriptome and Hormone Analyses Revealed Insights into Hormonal and Vesicle Trafficking Regulation among *Olea europaea* Fruit Tissues in Late Development

**DOI:** 10.3390/ijms21144819

**Published:** 2020-07-08

**Authors:** Beatriz Briegas, Jorge Corbacho, Maria C. Parra-Lobato, Miguel A. Paredes, Juana Labrador, Mercedes Gallardo, Maria C. Gomez-Jimenez

**Affiliations:** 1 Plant Physiology, University of Extremadura, Avda de Elvas s/n, 06006 Badajoz, Spain; bebrca04@alumnos.unex.es (B.B.); corsan.jorge@gmail.com (J.C.); mcarmenp@unex.es (M.C.P.-L.); mparedes@unex.es (M.A.P.); labrador@unex.es (J.L.); 2 Plant Physiology, University of Vigo, Campus Lagoas-Marcosende, s/n, 36310 Vigo, Spain; medina@uvigo.es

**Keywords:** cell wall, endomembrane trafficking, olive, plant hormone, fruit ripening, transcriptomic comparative

## Abstract

Fruit ripening and abscission are the results of the cell wall modification concerning different components of the signaling network. However, molecular-genetic information on the cross-talk between ripe fruit and their abscission zone (AZ) remains limited. In this study, we investigated transcriptional and hormonal changes in olive (*Olea europaea* L. cv Picual) pericarp and AZ tissues of fruit at the last stage of ripening, when fruit abscission occurs, to establish distinct tissue-specific expression patterns related to cell-wall modification, plant-hormone, and vesicle trafficking in combination with data on hormonal content. In this case, transcriptome profiling reveals that gene encoding members of the α-galactosidase and β-hexosaminidase families associated with up-regulation of RabB, RabD, and RabH classes of Rab-GTPases were exclusively transcribed in ripe fruit enriched in ABA, whereas genes of the arabinogalactan protein, laccase, lyase, endo-β-mannanase, ramnose synthase, and xyloglucan endotransglucosylase/hydrolase families associated with up-regulation of RabC, RabE, and RabG classes of Rab-GTPases were exclusively transcribed in AZ-enriched mainly in JA, which provide the first insights into the functional divergences among these protein families. The enrichment of these protein families in different tissues in combination with data on transcript abundance offer a tenable set of key genes of the regulatory network between olive fruit tissues in late development.

## 1. Introduction

In fleshy fruit, the fruit ripening and abscission are developmentally regulated and genetically programmed processes [1], whose induction depends on a complex interplay of a plant’s hormone content in addition to factors that alter the sensitivity of the tissues [2,3,4,5,6,7]. Knowledge of the mechanisms involved in fleshy-fruit ripening and abscission is essential for developing strategies to control yield. Although fully ripe fruit have marked physiological differences with respect to their abscission zone (AZ), both fruit ripening and abscission processes are the result of the cell wall modification, which involves a wide range of structural proteins and hydrolytic enzymes with distinct functions in the tissues (cell separation in the AZ and softening in the fruit), which concerns common and different components of the signaling network playing either direct or indirect roles in these processes. However, molecular-genetic information on the cross-talk between ripe fruit and their AZ is still limited.

Olive trees (*Olea europaea* L.) are one of the most economically important fruit trees worldwide for the oil of its fruit, and has high intra-specific genetic variation with a genome size of about 1800 Mb [8,9]. Until now, the study of gene function in the olive has been fundamentally advanced by the availability of whole genome sequences [10,11,12], while several transcriptomic, proteomic, and metabolic studies have been developed in olive fruit [13,14,15,16,17,18,19]. However, these studies concern only a few genotypes and little is still known about olive-fruit biology. In particular, information concerning the plant hormone composition of the olive fruit remains to be determined.

Events related to fruit ripening in some olive cultivars, such as cv. Picual trigger ripe fruit abscission [20]. In these cases, the patterns of abscission vary according to the cultivar [20]. In olive fruit, several AZs appear in the pedicel [21], even though only one AZ is specifically activated at a time at each stage of development [20,22]. In a previous study, we reported the comparison of the Picual fruit AZ transcriptomes at two different stages (pre-abscission vs. abscission) using the RNA-Seq technique. A total of 148 Mb of sequences (443,811 good-quality sequence reads) resulted and 4728 differentially-expressed genes were identified from these two samples [23]. Additionally, using 454 pyrosequencing technology, we also analyzed the overall transcriptional profile of Picual fruit pericarp at full ripening to significantly expand the olive transcript catalog [24]. In this cultivar, abscission of ripe fruit depends on the activation of the AZ located between the pedicel and fruit [22]. The excision of the AZ from olive fruit may lead to a contamination of the tiny AZ tissue with at least as much tissue from the surrounding pericarp/pedicel of the fruit. The comparison between AZ and adjacent tissues enable us to restrict the set of genes putatively related to the abscission process, and, in this sense, the results may provide worthwhile perspectives for the study of this process. Consequently, we compared the transcriptomes from AZ and pericarp tissues of olive ripe fruit in order to characterize the transcriptional factors enriched in differentially-regulated gene clusters, and presumably related to the abscission process, particularly ZF, bHLH, MADS-box, homeobox domain proteins, and bZIP families [24]. That represented the first effort to elucidate the divergences in transcriptional networks regulating the fruit abscission process in olives. Nevertheless, to date, no transcriptomic analysis has been conducted to determine the genetic changes associated with cell wall modification, plant-hormone, vesicle trafficking, and ion fluxes occurring among olive fruit and their AZ.

The aim of the present work was to analyze whole olive transcriptome changes in order to gain an understanding of the molecular mechanisms and hormonal control in olive fruit with respect to their AZ at the last stage of ripening when fruit abscission occurs. In particular, we focus in this study on the gene expression pattern involved in cell wall modification, plant hormone, vesicle trafficking, and ion fluxes. In addition, the hormonal composition was also examined in the olive fruit and their AZ at the last stage of ripening. These findings provide critical information for uncovering the differential hormonal and molecular genetic control between olive fruit tissues in late development.

## 2. Results and Discussion

### 2.1. Differential Abundance of Cell-Wall-Related Transcripts between Olive Fruit Tissues in Late Development

**Figure 1:**
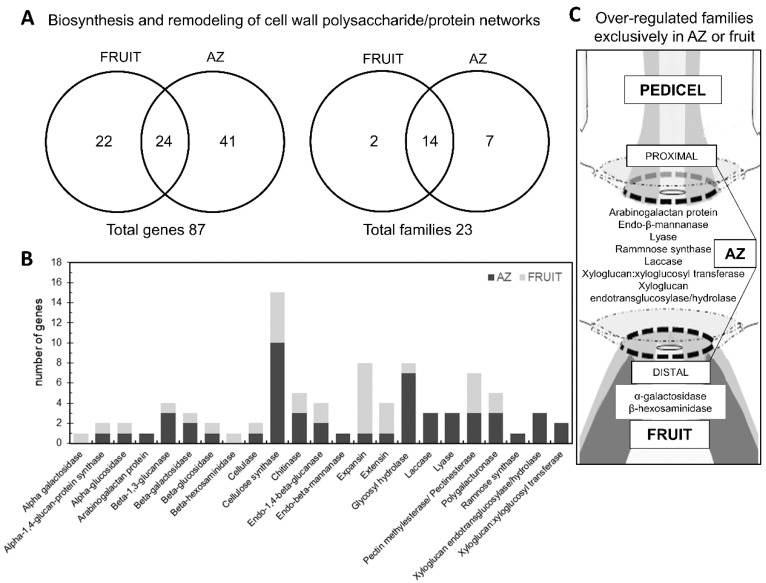
Differentially expressed cell wall-related genes and classification of cell wall families between olive AZ and fruit (pericarp) at the last stage of ripening (217 DPA). (**A**) Overlap of overexpressed fruit genes, and overexpressed AZ genes. This figure shows the number of transcripts and families that were specific and common for each tissue. (**B**) Comparison of significantly overexpressed transcripts (*p* < 0.01) between olive AZ (black) and fruit (gray) at the last stage of ripening. Number of transcripts related to the cell wall in each cell wall family. (**C**) Distribution of cell wall-related families that were specific for each olive tissue. Additional information on the cell wall-related genes is presented in Table S1.

Under natural conditions, olive of cv. Picual displayed around 92% abscission of ripe fruit at 217 days post-anthesis (DPA) [20]. For this study, two adjacent tissues of ‘Picual’ olive fruit, i.e., the fruit pericarp (epicarp and mesocarp) and the AZ, were selected at the last stage of fruit ripening (217 DPA), when fruit abscission occurs [22,24]. Recently, we have shown that the changes detected in AZ cell wall polysaccharides during ‘Picual’ fruit abscission are related to pectic polysaccharide de-esterification and solubilization [25], which is likely promoted by cell wall-associated pectin methyl esterases (PMEs) and polygalacturonases (PGs) enzymes. In the present study, to investigate ripening-abscission distinctions in ‘Picual’ olive, we compared the transcriptomes of olive fruit-pericarp vs. fruit-AZ at 217 DPA to restrict the set of cell wall genes presumably related to the abscission process. Our pyrosequencing-based approach enabled the identification of 87 differentially expressed genes (*p* < 0.01) in olive AZ compared to fruit at 217 DPA related to cell wall metabolism (Table S1, Figure 1A). Overall, 34 genes had peak read amounts (See Material and Methods) within the set of fruit-induced genes (‘fruit-enriched transcripts’), and 53 genes within the set of AZ-induced genes (‘AZ-enriched transcripts’) (Table S1).

Among the fruit-enriched genes, the most abundant proved to be a β-glucosidase protein (Table S1, Figure 1B). In olive, several members of the β-glucosidase family have been found in proteomic and transcriptomic studies [16,26]. Additionally, it has been proposed that β-glucosidase is a key enzyme in oleuropein catabolism [27,28,29,30]. However, in the ripe fruit, the well-represented families included expansin (EXP) (7 genes), cellulose synthase (CS) (5 genes), PME (4 genes), and extensin (EXT) (3 genes) proteins (Table S1, Figure 1), which suggests that these cell wall polysaccharide-degrading enzymes regulate olive-fruit softening. In particular, the fact that seven members of the EXP family showed differential expression in our study, suggests that this family may mediate cell wall loosening in olive ripe fruit.

The 10 most differentially overexpressed genes in the ripe fruit encoding cell wall proteins were one β-glucosidase (*Olea europaea*), α-EXP 8 (*Ricinus communis*), one EXP (*Diospyros kaki*), α-EXP3 (*Triphysaria versicolor*), one β-1,3-glucanase (*Nicotiana tabacum* NtEIG-E76), α-EXP11 (*Ricinus communis*), one PME (*Nicotiana benthamiana*), one EXP (*Vitis vinifera Vexp1*), one α-EXP (*Nicotiana tabacum*, Nt-EXPA3), and one EXT protein (*Catharanthus roseus* cyc17) (Table S1, Figure 1B).

Our team [23] and others [31,32,33,34,35,36,37,38,39] have reported AZ-enriched genes related to cell wall rearrangements during fruit abscission. In the present study, the well-represented classes in the AZ included CS (10 genes) and glycosyl hydrolase (GH) proteins (7 genes) (Table S1, Figure 1B), which indicates a key role for members of these families in abscission-associated cell wall changes. The 10 most differentially overexpressed genes in the AZ encoding cell wall proteins were one β-1,3-glucanase (*Olea europaea*, glu-4), one PG (*Olea europaea*), two laccase proteins *(Rosa hybrid cultivar* and *Populus trichocarpa,*), one glycosyl hydrolase GH 18 family protein (*Populus trichocarpa*), one chitinase protein (*Vitis vinífera*), two lyase proteins (*Ricinus communis*), one rhamnose synthase protein (RHM1, At1g78570), and one chitinase protein (*Vitis vinífera*) (Table S1, Figure 1B). This finding suggests that these types of enzymes may be required for complete cell separation of ripe fruit, and possibly for cell wall restructuring during AZ-cell separation.

Among the 34 fruit-enriched transcripts, 22 were exclusively expressed in fruit (Table S1). The 22 genes encode five EXP proteins, four CS proteins, four PME proteins, two EXT proteins, one cellulase or endo-β-1,4-D-glucanase protein, one α-1,4-glucan-protein synthase protein, one neutral α-glucosidase protein, one β-1,3-glucanase protein, one β-galactosidase protein, one α-galactosidase protein, one β-hexosaminidase protein, and one glycosyl hydrolase protein, which suggests that cell wall proteins from these families potentially have roles in mediating late events in ripe fruit. Similarly, among the 53 AZ-enriched transcripts (Table S1, Figure 1A), most of them (41) were expressed exclusively in the AZ compared to the ripe fruit. These genes encoding 10 CS proteins, four xyloglucan endotransglucosylase/hydrolase (XTH) proteins, four glycosyl hydrolase proteins, three lyase proteins, three PME proteins, two PG proteins, two laccase proteins, three endo-1,4-β-glucanase or cellulase proteins, two chitinase proteins, two β-galactosidase proteins, two β-1,3-glucanase proteins, one α-glucosidase protein, one arabinogalactan protein, one β-glucosidase protein, one EXP protein, and one EXT protein (Table S1, Figure 1B). These data agree with reports published in the expression of genes encoding for cell wall hydrolyzing enzymes associated with olive-fruit abscission [23]. Likewise, we recently reported that olive-fruit abscission was correlated with a reduced homogalacturonan methylesterification in the olive AZ [25], which suggests that these types of enzymes, 3 PME and 2 PG, may be required in regulating the level of homogalacturonan methylesterification during olive AZ cell separation necessary for abscission.

In this case, we report that, among all cell wall genes expressed differentially between the two olive tissues, only 22 genes were found to be expressed preferentially in the ripe fruit and 41 genes in the AZ (Table S1, Figure 1A). In this way, although two tissues containing members from several cell wall protein families, in each tissue, a clearly significant difference was found in the proportion of families (Table S1, Figure 1A). We hypothesize that the differences in the bulk of hydrolases and cell-wall-remodeling proteins between olive tissues may reflect differences in cell wall composition in different organs. Moreover, we identified different families of cell wall proteins that are expressed only in AZ (the arabinogalactan protein (AGP), laccase, lyase, endo-β-mannanase, ramnose synthase, XTH families) and families that are regulated only in fruit (the α-galactosidase and β-hexosaminidase families) (Table S1, Figure 1C). Based on expression analyses, we demonstrate that the AGP, laccase, lyase, endo-β-mannanase, ramnose synthase, and XTH families may have a function in cell wall modification related specifically to abscission. The enrichment of the cell wall protein families in different tissues in combination with data on transcript abundance offer a tenable set of cell wall genes that could be examined in future research.

### 2.2. Differential Hormonal Composition and Candidate Gene-Expression Patterns between Olive Fruit Tissues in Late Development

In olive, although the genomics data have identified hormone-related genes involved in fruit development and abscission processes [13,14,23], the hormonal composition of olive fruit remains to be determined. In previous works, we have reported on the polyamine levels in olive fruit during early development [29] as well as the 1-aminocyclopropane-1-carboxylic acid (ACC), an ethylene (ET) precursor, content [22], and the polyamine levels in the olive AZ during natural or induced olive-fruit abscission [20,40].

**Figure 2:**
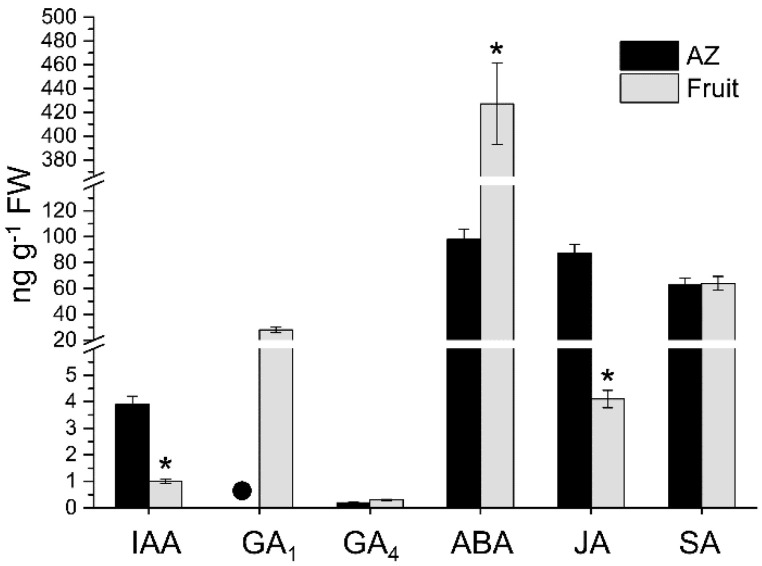
Profiles of IAA, GA_1_, GA_4_, ABA, JA, and SA levels measured from olive AZ and fruit (pericarp) at the last stage of ripening (217 DPA). Hormone levels not detected are indicated by a black dot (●). Data are the means ± SD of three biological replicates with three technical repeats each. Statistically significant differences based on unpaired Student’s *t*-test at *p* < 0.05 are denoted by an asterisk.

In the present work, the endogenous levels of indole-3-acetic acid (IAA), gibberellins (GAs), abscisic acid (ABA), jasmonic acid (JA), and salicylic acid (SA) in olive AZ and fruit during the last stage of ripening were measured (Figure 2), while the levels of bioactive free-base cytokinins (CKs) were too low to allow a reliable measurement. To our knowledge, this is the first report on the direct measurement of these hormones in olive AZ and fruit. Our results reveal that the total hormonal level was higher in the olive fruit (epicarp and mesocarp) in comparison with the AZ. The highest total hormone level in ripe fruit was due essentially to the highest ABA level. In both olive AZ and ripe fruit tissues, the most abundant hormone was ABA while the least abundant was GA_4_, but the ABA and GA_4_ levels were higher in the ripe fruit in comparison to the AZ, by 4-fold and 2-fold, respectively (Figure 2).

A high level of GA_1_, the 13-hydroxylated bioactive GA, but not GA_4,_ the 13-non-hydroxylated bioactive GA, was also detected in the ripe fruit, whereas GA_1_ was undetectable in the AZ, which indicates the higher activity in the 13-hydroxylated pathway. These results identify GA_1_ as the predominant bioactive GA in the olive reproductive organs. Low GA_4_ levels were detected in both olive tissues (Figure 2). By contrast, the profiling of hormone measurement revealed that the IAA and JA levels were higher in the AZ in comparison with the ripe fruit, by 4-fold, and 22-fold, respectively, whereas no significant differences were found in the SA level between olive AZ and ripe fruit (Figure 2). In olive ripe fruit, it bears noting that the most abundant hormone was ABA, which is followed by SA, while, in their AZ, the most abundant hormone was ABA, followed by JA (Figure 2). Altogether, these results indicate that high ABA and SA contents were found in both olive tissues in late development, and the presence of JA among the major hormones preferentially in the olive AZ.

**Figure 3:**
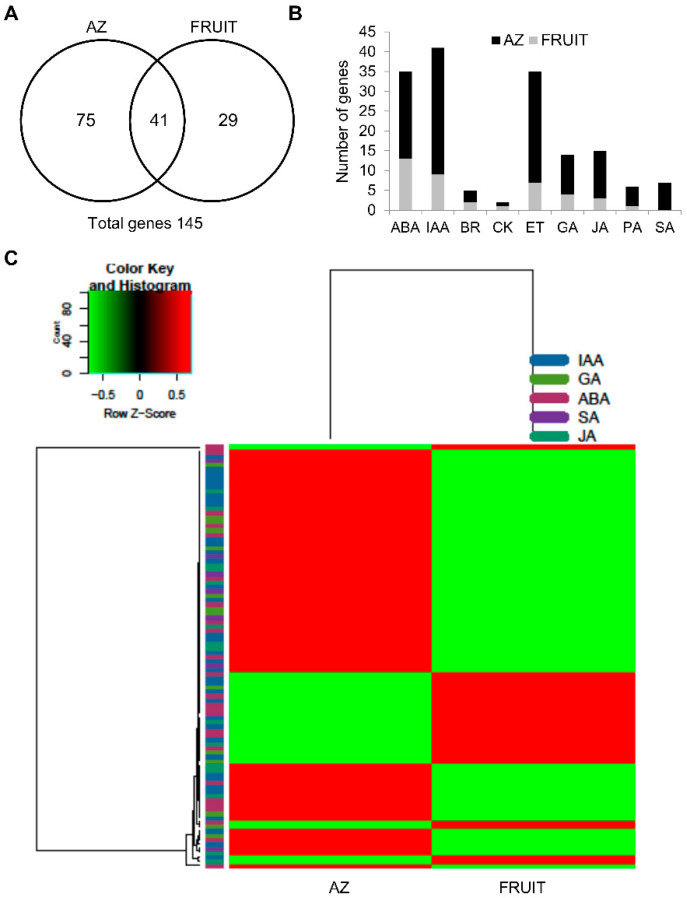
Differentially expressed hormone-related genes between olive abcission zone (AZ) and fruit (pericarp) at the last stage of ripening (217 DPA). (**A**) Overlap of overexpressed fruit genes, and overexpressed AZ genes. This figure shows the number of hormone-related transcripts that were specific and common for each tissue. (**B**) Comparison of significantly overexpressed transcripts (*p* < 0.01) between olive AZ (black) and fruit (gray) at the last stage of ripening. Number of transcripts related to the plant hormone in each tissue. (**C**) Heatmap of the expression of differentially expressed genes related to hormone metabolism and signaling in the indicated groups (IAA, GA_1_ + GA_4_, ABA, SA, JA levels) in olive AZ and fruit at the last stage of ripening. The gene expression of each sample was normalized using the mean expression for each condition. Then, the gene groups were defined in function of hormone relationship. Color codes for expression values are reported on the top. Additional information on the hormones-related genes is presented in Table S2 and Figure S1.

To determine how gene expression involved in hormone metabolism and signaling correlates with the accumulation of hormones in the olive tissues, we examined the transcriptomic profiling of genes associated with hormone metabolism and signaling in olive fruit and their AZ tissues in late development (217 DPA) through RNA-seq technology. Of 4,391 differentially expressed genes of our pyrosequencing-based approach (*p* < 0.01), 145 genes were related to plant-hormone metabolism and signaling (Figure 3A, Table S2) of which 36 showed a higher expression in the ripe fruit (fruit-enriched transcripts), while 109 were overexpressed in the AZ (AZ-enriched transcripts) (Table S2). Among the 145 genes, those related to auxin (36 genes), ET (35 genes), and ABA (25 genes) were the most represented, which is followed by those related to JA (15 genes) and GA (14 genes). Few genes related to SA (7 genes), polyamine (6 genes), brassinosteroid (BR, 5 genes), or CK (2 genes) were found (Figure 3B, Table S2). In particular, a comparison of the 145 hormone-related genes indicated that only 41 genes of these were common in both olive tissues, whereas 29 hormone-related genes were expressed exclusively in ripe fruit (fruit genes), and 75 hormone-related genes were expressed exclusively in their AZ (AZ genes) (Figure 3A, Table S2), which indicates a tissue-specific regulatory requirement for the expression of these hormone-related genes. Furthermore, we explored the tentatively link of the level of the plant hormones detected to the gene expression involved in their metabolism and signaling in both olive tisssues (Figure 3C, Figure S1). Taking together the expression analyses and the hormone levels in both olive tissues, we built a heat map referring to IAA, GA, ABA, JA, and SA (Figure 3C).

**Figure 4:**
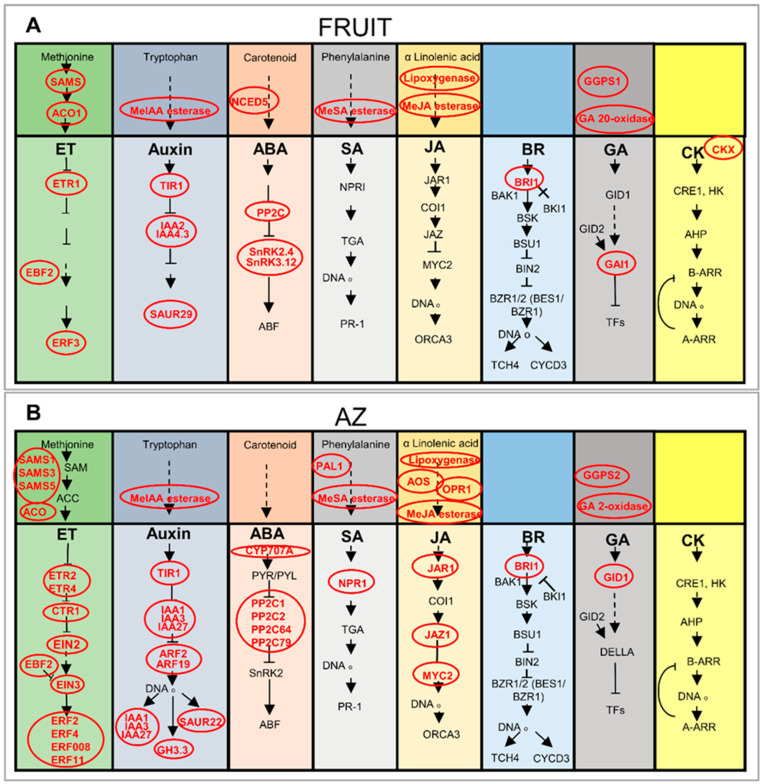
Simplified schematic representation of the hormone metabolism and signaling pathways in AZ and fruit at the last stage of ripening in olive (217 DPA). (**A**) Fruit-enriched genes encoding various hormone proteins at 217 DPA (*p* < 0.01). (**B**) AZ-enriched genes encoding various hormone proteins at 217 DPA (*p* < 0.01). Genes with elevated mRNA levels are in red typeface (ET = Ethylene). Additional information on the hormone-related genes is presented in Table S2.

Figure 4 illustrates the biosynthesis and signaling of plant hormones, and reflects that steps in the pathways of ET, auxin, ABA, SA, JA, BR, GA, and CK appear to be transcriptionally regulated between olive ripe fruit and their AZ tissues. Notably, among the 145 genes related to the plant-hormone, those related to ABA (9 genes) and auxin (9 genes) were the most represented in ripe fruit, whereas those related to ET (28 genes) and auxin (27 genes) were the most represented in AZ (Table S2). *NCED5*, which is a transcript associated with ABA biosynthesis, was expressed exclusively in ripe fruit (Figure 4A, Table S2). NCED is involved in catalyzing the rate-limiting step in ABA biosynthesis [41]. In addition, other transcripts involved in ABA catabolism such as cytochrome P450 *CYP707A*, which encodes ABA 8′-hydroxylase, was expressed exclusively in AZ (Table S2). This is consistent with the highest ABA levels detected in olive ripe fruit compared to AZ (Figure 2). Previously, the downregulation of *NCED5* and upregulation of *CYP707A* genes during abscission in the olive-fruit AZ has been demonstrated [23], which suggests local transcriptional control of the ABA biosynthesis and catabolism rather than transport from other tissues. In the case of auxin, transcripts involved in IAA transport, such as two transcripts encoding auxin influx carrier-like protein 1 (LAX1) and 2 (LAX2), are exclusively overexpressed in olive AZ (Table S2). This suggests a role in regulating auxin influx and in maintaining auxin sink-strength in this tissue in a manner similar to that of its arabidopsis and tomato orthologs, AtLAX3 and SlLAX3, which have been shown to create cell-specific auxin sinks [42,43]. This is consistent with previous studies on abscission in which genes encoding for protein homologs of this family were found to be up-regulated [23,33,35,44]. In addition, two different transcripts encoding auxin efflux carrier are overexpressed in the AZ or in fruit (Table S2), which indicates altered auxin distribution in these tissues in late development.

In the case of SA, the mRNA for PAL1, which are associated with the SA biosynthesis, was exclusively up-regulated in AZ (Figure 4B, Table S2), but SA levels were not significantly different between AZ and ripe fruit, whereas transcripts for some GA biosynthetic enzymes components such as GA 20-oxidase (GA20ox), involved in a late GA biosynthetic step, were enriched exclusively in ripe fruit (Figure 4A, Table S2) and consistently with the highest GA levels detected in olive ripe fruit compared to AZ. Conversely, *GA2ox5*, which is a transcript involved in the deactivation of bioactive GAs, was expressed exclusively in AZ (Figure 4, Table S2), which implies that GA2ox dominantly functions in the AZ, while different transcripts, *GGPS1* and *GGPS2*, for geranylgeranyl pyrophosphate synthase (GGPS), which is involved in an early GA biosynthetic step, were overexpressed in olive fruit (*GGPS1*) or AZ (*GGPS2*) (Figure 4, Table S2). The expression of early GA synthesis genes at a low level in the AZ implies that GA may not be actively synthesized in the AZ. These results suggest that GA20ox is the major late-step enzyme responsible for the high-level accumulation of GA_1_ detected in ripe fruit, whereas the complete absence of GA_1_ observed in the AZ may be attributed to the expression of the *GA2ox* gene. Moreover, of the five differentially expressed genes involved in JA biosynthesis, four genes show increased transcript abundance in AZ compared to fruit in good agreement with the highest JA content in the AZ (Figure 4B, Table S2). These results indicate that JA may be actively synthesized in the AZ.

On the other hand, several transcripts related to hormonal metabolism were also differentially expressed between olive ripe fruit and AZ tissues, such as genes encoding putative methylesterases, which can hydrolyze MeJA, MeSA, and MeIAA (Table S2, Figure 4). However, transcript levels for some auxin-conjugating enzymes, such as GH3.3, were found to be exclusively expressed in AZ (Table S2), which is consistent with previous studies where *GH3.3* expression is induced during ripe fruit abscission in olives and melons [23,44]. Bioactive forms of CKs are, in turn, deactivated by CK oxidase/dehydrogenase (CKX), and, thereby, regulates the amount of bioactive CK [45]. In the present study, *CKX* gene expression is found exclusively in the ripe fruit, which suggests that the deactivation of CK may actively occur in ripe fruit (Figure 4A, Table S2).

Additionally, our data indicate that numerous genes encode key players related to the ET pathway (Table S2, Figure 4). Both ripe fruit and AZ tissues are apparently characterized by an active ET biosynthesis at the transcriptional level. In particular, one S-adenosylmethionine synthase (*SAMS*) gene and one ACC oxidase (*ACO1*) gene were overexpressed in the ripe fruit (Figure 4A, Table S2), while three different *SAMS* genes (*SAMS1*, *SAMS3*, and *SAMS5*) were up-regulated in the AZ compared to the ripe fruit, and one different *ACO* gene was expressed exclusively in the AZ (Figure 4B, Table S2). Previously, our pyrosequencing data indicated higher olive AZ expression of ET-related genes for the SAMS, ACS, and ACO during ripe fruit abscission [23].

The present analysis also detected the differential expression of many genes involved in plant-hormone signaling between ripe fruit and AZ tissues (Figure 4, Table S2). Genes specifying auxin signaling components (IAA2, IAA4.3, and SAUR29) as well as ABA signaling components (PP2C, SnRK2.4, and SnRK3.12) had also raised RNA levels in ripe fruit (Figure 4B, Table S2), while others (IAA1, IAA3, IAA27, ARF2, ARF19, SAUR22, GH3.3, PP2C1, PP2C2, PP2C64, and PP2C79) were overexpressed in AZ. In particular, the specific expression of some *ARF* (*ARF2* and *ARF19*) and *IAA/AUX* (*IAA1*, *IAA3*, and *IAA27*) genes in the AZ suggests that ARF2 and ARF19 are involved in the abscission of ripe fruit. Functional studies of ARF2, ARF1, ARF7, and ARF19 suggest that these transcription factors act with partial redundancy to promote senescence and floral abscission [46]. Similarly, our previous data indicate that two families of early auxin-responsive genes, IAA27 and GH3.3, which contain a binding motif to the ARF transcription factor, are up-regulated during induction of ripe fruit abscission, whereas one *SAUR1* gene is down-regulated during olive ripe fruit abscission [23]. In addition, transcript abundance of putative ABA signaling gene, *ABA-INSENSITIVE 2* (*ABI2*), proved significantly lower in ripe fruit compared to AZ (Table S2). *ABI2* encodes a member of the 2C class of protein serine/threonine phosphatases (PP2C), which is a negative regulator of ABA responses [47]. Among fruit-enriched transcripts, *SnRK2.4* and *SnRK3.12*, were exclusively expressed in ripe fruit (Table S2). Thus, these differences in auxin-related and ABA-related responses suggest that auxin signaling is more active in the AZ than in ripe fruit, while the ABA signaling is more active in the ripe fruit than in the AZ. In the case of GA, the mRNA for GAI1 (DELLA protein), was also up-regulated in ripe fruit, which suggested that GA signaling is negatively regulated by GAI in ripe fruit. In contrast, some transcripts for SA and JA signaling markers were down-regulated (*NPR1*, *JAR1*, *JAZ1*, and *MYC2*) in ripe fruit (Figure 4A, Table S2), whereas they were overexpressed in AZ (Figure 4B, Table S2). This suggested that NPR1, JAR1, JAZ1, and MYC2 may have functions in the AZ cell separation.

The gene profiling data also showed that *ETR1* and *ERF3* transcripts, which are markers for the ET response, were up-regulated in ripe fruit (Figure 4A, Table S2), whereas others (*ETR2*, *ETR4*, *CTR1*, *EIN2*, *EIN3*, *ERF2*, *ERF4*, *ERF008*, and *ERF11*) were up-regulated in the AZ (Figure 3B, Table S3). This is consistent with previous studies where the expression of some *ETR2*, *ETR4*, *CTR1*, *EIL2 (EIN3/EIL*), and *ERF4* genes are induced during ripe fruit abscission [22,23]. The ERFs are the main mediators of ET-dependent gene transcription. In apple, ERF3 promotes, whereas ERF2 represses, the expression of *ACS1* [48]. In the tomato genome, 27 ERFs show enhanced expression at the onset of ripening while 28 ERFs display a ripening-associated decrease in expression, which suggests that different ERFs may have contrasting roles in fruit ripening [49]. In this case, our results suggest that different ERFs could be involved in triggering the transcriptional cascade in the AZ (ERF2, ERF4, ERF008, and ERF11) or in the ripe fruit (ERF3). Thus, our study demonstrates the induction of the ET signaling pathway in both olive tissues via different components.

Up-regulation of BR signaling was unexpected in both olive tissues (Figure 4, Table S2). The present work shows that different transcripts encoding BRI1 receptor kinase were up-regulated in AZ and ripe fruit (Figure 4, Table S2), which suggests that the up-regulation of receptor BRI1 may be required for complete AZ cell separation in olives.

**Figure 5:**
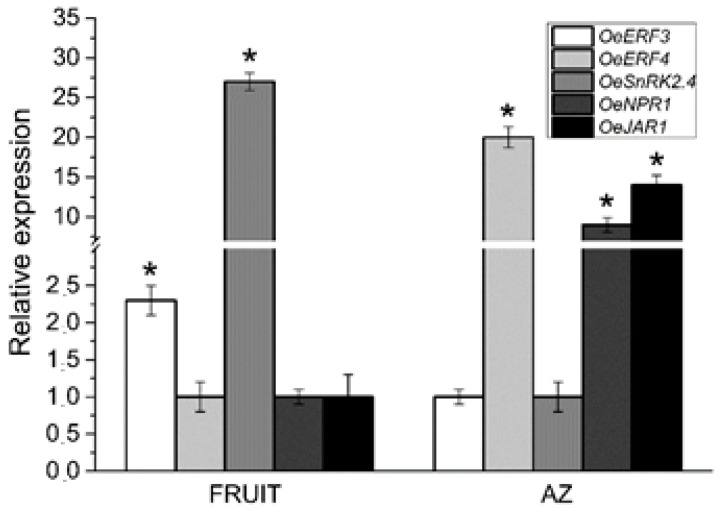
Expression of *OeERF3, OeERF4*, *OeSNRK2.4*, *OeNPR1*, and *OeJAR1* in AZ and fruit at the last stage of ripening (217 DPA) in olives. Data are the means ± SD of three biological replicates with three technical repeats each and were determined by qRT-PCR normalized against *Olea europaea* ubiquitine. Statistically significant differences based on unpaired Student’s *t*-test at *p* < 0.05 are denoted by an asterisk.

We used qRT-PCR to verify induction of the ET and ABA signaling pathways in both tissues via different components, and up-regulation of the SA and JA signaling pathways in the AZ. In addition, we examined the expression of this set of hormone response genes (*OeERF3, OeERF4*, *OeSNRK2.4*, *OeNPR1*, and *OeJAR1*) by qRT-PCR in olive AZ and ripe fruit (Figure 5). The qRT-PCR analysis confirmed the enrichment of *OeERF3*, and *OeSNRK2.4* genes in ripe fruit and the enrichment of *OeERF4*, *OeNPR1*, and *OeJAR1* genes in the olive AZ.

### 2.3. Vesicle Trafficking Differential Gene Expression between Olive Ripe Fruit and AZ

**Figure 6:**
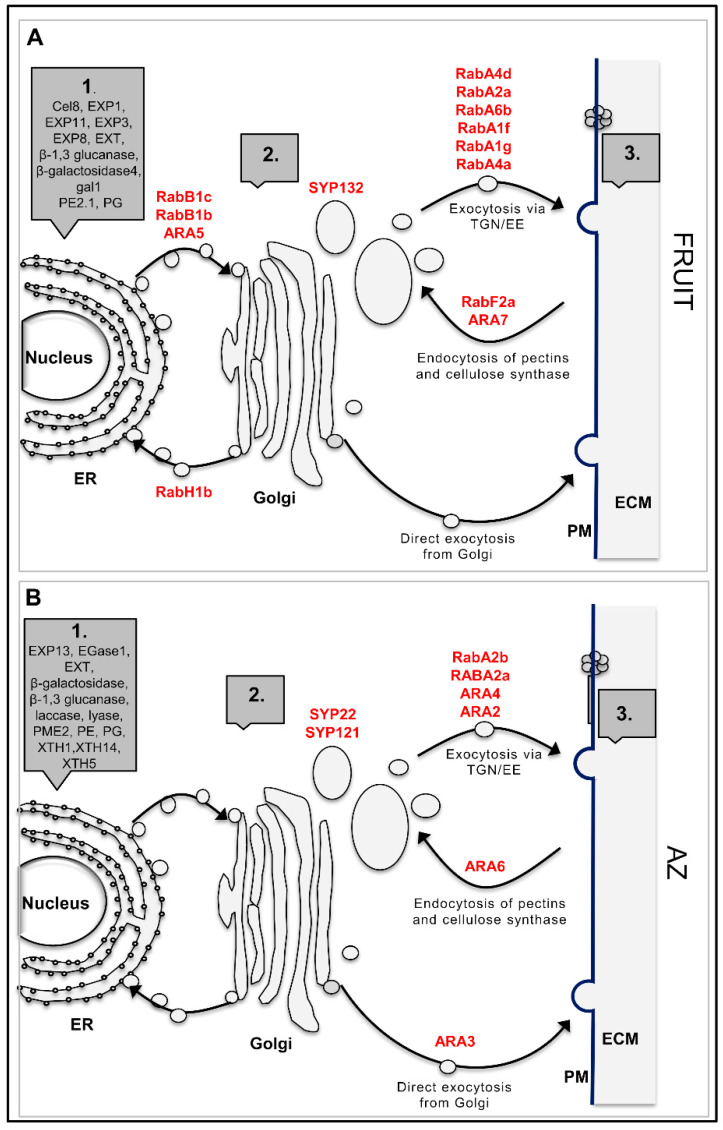
Simplified schematic representation of the trafficking pathways to and from the cell wall of olive fruit (**A**) and AZ (**B**) at the last stage of ripening. The Rab-GTPases likely involved at each step are indicated in red (up-regulated). 1. Synthesis of proteins in endoplasmic reticulum (ER). 2. Synthesis of matrix polysaccharides and assembly of proteins in Golgi and TGN/EE (the trans-Golgi network and early endosomal compartments). 3. Modification of wall elements by secreted enzymes. Pathways to and from the vacuole have been omitted for simplicity. Additional information on the vesicle-trafficking-related genes is presented in Table S3. (ECM: equivalent to the “cell wall” or “apoplast”. PM: plasma membrane).

Previously, we have reported that endocytosis, visualized by staining with fluorescent dye FM4-64, was strongly stimulated in AZ during olive ripe fruit abscission [50], which suggests that endomembrane trafficking likely modulates cell wall modifications during olive fruit abscission. Deposition changes in cell-wall material involve vesicle formation and transport, which is reflected by a high number of up-regulated genes found in ripe fruit and AZ tissues in olives (Figure 6, Table S3). In particular, the Rab GTPase is a key component of the membrane trafficking machinery that regulates the targeting and tethering of trafficking vesicles to target compartments by acting as a molecular switch cycling between active and inactive states [51,52,53]. However, little is known about Rab-GTPases comparing the transcriptomic responses between fruit and their AZ. In this scenario, among the 24 Rab-GTPases identified in our analysis, 14 Rab-GTPases were up-regulated in ripe fruit and 10 Rab-GTPases in fruit AZ (Figure 6, Table S3), which indicates that at least some members of Rab-GTPases play major roles in secretion and/or recycling of cell wall components in these olive-fruit tissues in late development.

We identified different classes of Rab-GTPases genes that are regulated only in the AZ (the arabidopsis RabC, RabE, and RabG clades) in both AZ and fruit (the arabidopsis RabA and RabF clades) or only in the fruit (the arabidopsis RabB, RabD, and RabH clades) (Table S3). Five Rab11 (corresponding to the arabidopsis RabA clade, gene Identifier AT5G47960.1, AT1G09630.1, AT5G60860.1, AT3G15060.1, and AT5G65270.1 putative orthologs), three Rab2 (corresponding to RabB, AT4G35860.1, AT1G02130.1, and AT4G17170.1), one Rab1 (corresponding to RabD, AT1G02130.1), two Rab5 (corresponding to RabF, AT5G45130.1, and AT4G19640.1), and two Rab6 (corresponding to RabH, AT1G18200.1, and AT2G44610.1) genes from olives showed a higher expression in ripe fruit (Figure 6A, Table S3) compared to the AZ, while, for four Rab11 (corresponding to RabA, putative ortholog AT1G06400.1, AT2G43130.1, AT1G09630.1, and AT1G07410.1), one Rab18 (corresponding to RabC, AT5G03530.1), one Rab8 (corresponding to RabE, AT3G46060.3), one Rab5 (corresponding to RabF, AT3G54840.1), and two Rab7 (corresponding to RabG, AT3G18820.1, and AT4G09720.1) genes from olives, a higher number of average reads per sample was detected in the AZ (Figure 6B, Table S3).

Of these classes of Rab-GTPases, two (Rab11/RabA and Rab18/RabC) expressed preferentially in apple, peach, and tomato fruits during the final ripening stages [52,54,55]. Therefore, these Rab GTPase classes apparently share major trafficking elements related to the cell wall modification in ripe fruit. On the other hand, the Rab5/RabF and Rab7/RabG classes in olives have been determined to be specific to ripe fruit abscission [23]. Particularly, the up-regulation of the genes *RabA2A*, *RabA2B*, and *RabA5C* in the AZ of olives during ripe fruit abscission suggests that at least some RAB11 genes play a key part in recycling and/or secretion of cell-wall components in the process of ripe fruit abscission [23]. This contention agrees with our prior results demonstrating the up-regulation of genes *RabA2A*, *RabA2B*, and *ARA6* as well as the down-regulation of the *RabH1B* gene in melon AZ during ripe fruit abscission [25]. Thus, these constitute different classes of Rab-GTPase protein and, therefore, would presumably regulate either transport to the plasma membrane and the cell wall, exocytosis from the trans-Golgi network, or vacuolar trafficking of these tissues in late development.

Notably, our data reveal that the ARA6 (RabF1) gene is expressed exclusively in the AZ (Figure 6B, Table S3) in which this is consistent with previous findings, where the expression of some ARA6 genes was induced in the AZ during fruit abscission [23,44], whereas the ARA7 gene is exclusively expressed in the fruit (Figure 6A, Table S3). This indicates that transcript levels of ARA6 and ARA7 genes regulate endocytic and vacuolar trafficking pathways, which are regulated in a tissue-specific manner.

In addition, the synthaxin genes, SYP121 and SYP132 (t-SNARE family), related to ABA-responsive secretion [56,57] were also up-regulated exclusively in AZ and ripe fruit, respectively. In relation to this, synthaxin SYP121, involved in the regulation of SA and JA [58], is upregulated during ripe fruit abscission in olives and melons [23,44]. Moreover, the synthaxin SYP22, which is required for vacuolar assembly [59], is also up-regulated in the AZ (Figure 6B, Table S3). Previous work has shown that a strong induction of *ARA6* in the AZ during olive fruit abscission is parallel with the upregulation of *SYP121* and *SYP22* syntaxins, which implies the involvement of ARA6 in the trafficking pathway to modulate ripe fruit abscission in olives, and a possible involvement of SYP121 and SYP22 in a common process [23]. ARA6 putatively act in a trafficking route that counteracts endocytic trafficking from the endosomes to the vacuoles, where SYP22 fulfills a positive regulatory role. On this basis, it might be asked whether this SYP132 protein plays an essential part together with some of the different up-regulated Rab-GTPases in olive fruit for the transport of proteins and membrane through the endomembrane system to their destination, and whether this transport plays a critical role in mediating plant-hormone signals in ripe fruit.

In this study, some members of Rho GTPase family were also identified (Table S3). One gene homologous to MIRO1, which has evolved to regulate mitochondrial trafficking, and one gene homologous to ARAC5 were exclusively transcribed in the AZ, whereas one gene homologous to RAC3 is exclusively transcribed in ripe fruit. Additionally, our data demonstrate that one member of Ran GTPase family homologous to Ran3 [60] involved in the nuclear translocation of proteins in arabidopsis, is up-regulated in the AZ (Table S3), as previously reported for ripe fruit abscission [23]. This strengthens the possibility that that they may help mediate nucleocytoplasmic transport during fruit abscission signaling. Of particular interest is also one member of Sar1 GTPases family, homologous to SAR2 [61], which was expressed exclusively in ripe fruit (Table S3).

Therefore, our results indicate that genes encoding members of small-GTPase Arf and Sar families were exclusively transcribed in ripe fruit. By contrast, small-GTPase genes encoding members of Ran family were exclusively transcribed in the AZ. These results raise the possibility that the Rabs, Rhos, and Ran families of small-GTPases may act in vesicle trafficking in the AZ, while ripe fruit is enriched in the Rabs, Rhos, Arfs, and Sar1 families of small-GTPase, which suggests that Rabs-GTPase and Rhos-GTPase families may act in vesicle trafficking in both olive tissues in late development. Moreover, three classes of the Rab family, i.e., RabB, RabD, and RabH, have been found to be preferentially expressed in the ripe fruit, whereas the classes RabC, RabE, and RabG have been found to be preferentially expressed in olive AZ. Other genes noticeably present in AZ and ripe fruit involved in vesicle trafficking encode V-type ATPases, called midasins (Table S3), which were involved in trafficking from the trans-Golgi Network to the central vacuole. Thus, these data provide novel information about small-GTPases in late development, which suggests that vesicular trafficking may regulate fruit and AZ cell wall modifications in late development.

### 2.4. Global Expression Profiling of Transport Protein Genes

**Figure 7:**
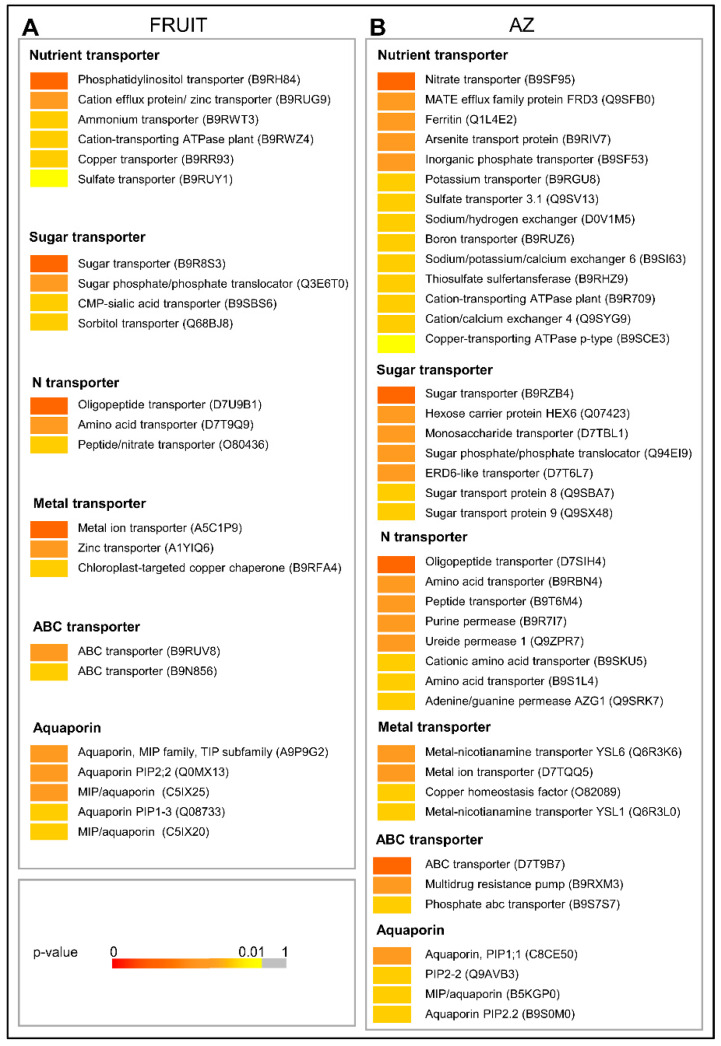
Expression profiling of (**A**) fruit-enriched or (**B**) AZ-enriched genes related to transport as reconstructed from the pyrosequencing transcriptomes. Sequence transcripts showing significant variations (*p* < 0.01). *p*-values are visualized in a color-code scale. Additional information on the transport-related genes is presented in Table S4.

Of 4,391 differentially expressed genes, 138 genes related to transport were differentially expressed in olive AZ compared to ripe fruit (*p* < 0.01). Of these genes, 48 genes had peak read amounts in fruit (the set of fruit-induced genes), and 90 genes in AZ (the set of AZ-induced genes) tissues (Figure 7, Table S4). Thus, the majority of these were induced in the AZ (Figure 7, Table S4).

Among the 48 genes enriched in ripe fruit, 31 were exclusively expressed in fruit (Table S4). The 31 genes encode three sugar transporter, four N transporter, three aquaporin, four nutrient transporter, two metal ion transporter proteins, and one ATP-binding cassette (ABC) transporter family protein, whereas our analyses have revealed that 90 transport-related genes were induced in the AZ, 75 genes (11 sugar transporter, 20 N transporter, three aquaporin, 22 nutrient transposter, nine metal ion transporter proteins, and 10 ABC transporter family proteins) are exclusively expressed in the AZ (Figure 7, Table S4), which indicates that transporters play special roles in the tissue-specific characteristics of olive ripe fruit. Among these latter proteins, one ERD6 sucrose transporter was upregulated during ripe fruit abscission, whereas one hexose carrier protein HEX6 was downregulated during ripe fruit abscission in olives [23]. In addition, of AZ-enriched genes related to nutrient transport in our analysis, three are associated with sodium/hydrogen exchangers, three cation-transporting ATPases, two nitrate transporters, two potassium transporters, two phosphate transporters, one MATE citrate transporter, one sulfate transporter, one boron transport, and one arsenite transport protein, whereas three cation-transporting ATPases, three 2-oxoglutarate/malate translocator proteins, two copper transporters, two sulfate transporters, and one phosphatidylinositol transporter were induced in ripe fruit, which indicated that most of these transporter genes were preferentially expressed in AZ (Figure 7, Table S4). Thus, function enrichment analysis of these genes at the tissue level shows that there are more enriched functions in the AZ than in the pericarp of olive fruit at the last stage of ripening when fruit abscission occurs.

No previous study has been focused on the nutrient transport between olive fruit tissues. In Citrus (*Citrus* spp.), it has been shown that the different flesh-rind transport of nutrients and water due to the anatomic structural differences among citrus varieties might be an important factor that influences fruit senescence behavior [62]. Our results appear to reflect differences in nutrient transport between AZ and pericarp tissues of olive fruit at the last stage of ripening. For example, the AZ transport results mainly accomplished by the gene activity of nitrate, boron, potassium, and phosphate transporters, and the fruit-pericarp transport by copper transporter, whereas the transport of sulfate and calcium are associated with both olive tissues in late development. Therefore, our results corroborate previous works identifying nitrate and boron transporters as being induced in olives and melon fruit AZ during abscission [23,44]. Based on our data, we suggest that, for the AZ of ripe fruit, these changes could be associated with nutrient re-mobilization prior to abscission. Previous studies have shown that increased ethylene production may be involved in modulating nitrate transporters [63] and nitrate metabolism [64] at high nitrate levels. In the plant, nitrate can serve as a signaling molecule in an array of physiological processes and environmental responses [65,66], but the role in abscission played by nitrate has received little attention. Further studies are needed to explore the exact roles of these transporters in regulating olive fruit abscission.

Among differentially-expressed genes, we also detected 26 genes related to nitrogen transport and enriched in AZ, different cationic amino acid transporters, amino acid transporters, oligopeptide transporters, purine permeases, one PTR2/POT transporter, peptide transporters, and one ureide permease (UPS1) among others (Table S4). Meanwhile, three other amino acid transporters, seven other oligopeptide transporters, and one peptide/nitrate transporter (At2g38100) were overexpressed in ripe fruit (Table S4). ABC transporter family proteins were also accumulated abundantly in the AZ (Figure 7, Table S4). Among these, six encode multi-drug resistance-associated protein, known as the ABC transporter of the B class (ABCB) protein, which functions in auxin transport across plant species [67,68,69]. At the same time, two other ABC transporters are overexpressed in fruit. Thus, the present study provides information for identifying candidate channel and transporter genes possibly involved in a transporter-mediated transportation process from the fruit to the AZ.

In summary, our comprehensive analyses of differential gene expression combined with analysis of differential hormonal composition revealed the unique and common gene signatures between olive fruit tissues in late development. Data reported in this case represent a significant contribution to the elucidation of transcriptional networks related to cell wall modification, plant hormone, vesicle trafficking, and ion fluxes in the AZ and the pericarp of olive fruit at the last stage of ripening. Particularly noteworthy are data related to hormones that indicate the complexity of the role played by these compounds in these adjacent tissues. Furthermore, our data reveal that the olive ripe fruit-pericarp was found to be rich in ABA, SA, and GA_1_, whereas the fruit AZ at the last stage of ripening, when fruit abscission occurs, was enriched mainly in JA. In addition, by qRT-PCR analysis, we confirmed the mRNA-Seq results for five hormone signaling genes, and the induction of the ET signaling pathway in both olive tissues via different components. The transcriptomic patterns in AZ and pericarp of olive ripe fruit offer new insights about hormonal and vesicle trafficking regulation potentially involved in cell wall modifications.

## 3. Materials and Methods 

### 3.1. Plant Material

In an orchard near Badajoz (Spain), 20-year-old olive trees (*Olea europaea* L. cv. Picual) grown under drip irrigation and fertirrigation (irrigation with suitable fertilizers in the solution) were studied. Picual olive flowers were tagged on the day of pollination and the fruit pericarp (fruit mesocarp and epicarp) and fruit-AZ samples were collected from olive fruits subsequently harvested during the last stage of ripening (217 DPA) at which time they abscise [22,23,24]. The fruit AZs, located between the pedicel and fruit, were manually dissected from longitudinal sections of the samples with a razor blade into pieces to a maximum width of 1 mm on each side of the abscission fracture plane (Figure S2). Fresh samples (fruit-pericarp and fruit-AZ at 217 DPA), using 300 fruits, were immediately frozen in liquid nitrogen and stored at −80 °C for RNA isolation. For examination of the proximal and distal fracture planes of the fruit AZ by scanning electron microscopy (SEM), following critical-point drying, tissues were mounted onto steel stubs, coated with gold-palladium, and viewed using a LEO 1430 VP scanning electron microscope [20,44].

### 3.2. RNA Isolation

Total RNA and cDNA synthesis were extracted and purified from fruit-pericarp (mesocarp and epicarp) and-AZ tissues at 217 DPA as detailed in Reference [24].

### 3.3. Library Preparation for Pyro-Sequencing

Three micrograms of each cDNA sample were nebulized to produce fragments of a mean size between 400 and 800 bp. Preparation of cDNA fragment libraries and emulsion PCR conditions were performed as described in the Roche GS FLX manual. Pyro-sequencing was performed on a Roche Genome Sequencer FLX instrument (454LifeScience-Roche Diagnostics, http://www.454.com/) at Lifesequencing S.L. (Valencia, Spain). Trimming and assembly of pyro-sequenced reads and annotation were performed as described [24].

### 3.4. Quantification of the Expression Levels

The reference proteins were proteins representative of UniRef90 clusters. This strategy fixed a minimum similarity distance between reference proteins and was the basis of our clustering of isotigs for obtaining unigenes and quantifying their expression levels. The name of each unigene was inferred from the name of the UniRef90 representative proteins that annotated each unigene. We quantified the expression for these unigenes, defined here as clusters of isotigs annotated by the same reference protein. The number of reads assigned to each isotig was calculated by taking into account that the reads of each contig were counted only one at a time. Given that isotigs represent transcribed isoforms, different isotigs sharing some contigs could possibly be clustered within the same unigene. In those cases, the reads of each contig was counted only one time. The normalization of the absolute values of the number of reads was done based on Reference [70]. We obtained the RPKM (Reads Per Kilobase of exon model per Million mapped reads). In this case, we used the length of the reference protein in nucleotides since we were working without a reference genome and then without exon models. This normalization allows the comparison of the expression values between unigenes from the same or from different simples [24].

### 3.5. Differential Expression Analysis

The method used for the analysis of differential expression in this work was edger [71], a Bioconductor package for differential expression analysis of digital gene-expression data able to account for biological variability. EdgeR models count data using on overdispersed Poisson model, and use an empirical Bayes procedure to moderate the degree of over-dispersion across genes. For the analysis of the differential expression with Edge R, the input was a table of counts with rows corresponding to genes/proteins and columns to samples. EdgeR models the data as negative binomial (NB) distributed, Ygi~NB (Mipgj, Φg) for gene g and sample i. In this case, Mi is the library size (total number of reads), Φg is the dispersion, and pgj is the relative abundance of gene g in experimental group j to which sample i belongs. The NB distribution reduces to Poisson when Φg = 0. In this work, an isotig was considered differentially expressed when it exhibited a highly significant difference in read abundance at *p* < 0.01. The heatmap was drawn using gplots library of the R platform and cluster evaluation defined using a Silhouette estimator [72].

### 3.6. Quantification of Plant Hormones

A pool of 100-mg fresh weight of samples was used for each measurement, and divided into three independent biological replicates. Quantification of plant-hormones was performed as described by Reference [73]. Aliquots of lyophilized material were extracted with 80% methanol-1% acetic acid. Deuterium-labeled hormones (purchased from Prof. L Mander- Canberra, OlChemim Ltd-Olomouc, or Cambridge Isotope Lab- Andover): [17,17-^2^H]GAn, [^2^H_5_]IAA, and [^2^H_6_]ABA were added as internal standards for quantification of SA and ABA. For quantification of JA, the compound dhJA was used instead. For collecting the acids fraction containing SA, ABA, and JA, the extracts passed consecutively through HLB (reverse phase), MCX (cationic exchange), and WAX (ionic exchange) columns (Oasis 30 mg, Waters). The final residue was dissolved in 5% acetonitrile-1% acetic acid, and the hormones were separated by reverse phase UPHL chromatography (2.6 µm Accucore RP-MS column, 100 mm length × 2.1 mm i.d., ThermoFisher Scientific) with a 5% to 50% acetonitrile gradient. The hormones were analyzed by electrospray ionization and targeted-SIM using a Q-Exactive spectrometer (Orbitrap detector, ThermoFisher Scientific, Spain). The concentrations of hormones in the extracts were determined using embedded calibration curves and the Xcalibur 4.1 SP1 build 48 and TraceFinder programs.

### 3.7. Quantitative RT-PCR

Total RNA (2 μg) was reverse-transcribed with random hexamers and Superscript III (Invitrogen), according to the manufacturer’s instructions. Purified cDNA (2 ng) was used as a template for quantitative RT-PCR (qRT-PCR). qRT-PCR assays were performed with gene-specific primers (Table S5). The cDNA was amplified using SYBRGreen-PCR Master kit (Applied Biosystems, Foster City, CA, USA) containing an AmpliTaq Gold polymerase on an iCycler (BioRad Munich, Germany) by following the supplier’s protocol. Samples were subjected to thermal cycling conditions of DNA polymerase activation at 94 °C, 45 s at 55 °C, 45 s at 72 °C, and 45 s at 80 °C. A final elongation step of 7 min at 72 °C was performed. The melting curve was designed to increase 0.5 °C every 10 s from 62 °C. The amplicon was analyzed by electrophoresis and sequenced once for identity confirmation. In addition, qRT-PCR efficiency was estimated via a calibration dilution curve and slope calculation. Expression levels were determined as the number of cycles needed for the amplification to reach a threshold fixed in the exponential phase of the PCR (CT). The data were normalized for the quantity of *Olea europaea* ubiquitin (*OeUB*) genes [74]. Duplicates from three biological replicates were used in two independent experiments.

### 3.8. Data Availability

The complete set of 454 sequences will be deposited in GenBank upon publication. The data set can also be obtained from the authors via FTP upon request.

## Supplementary Materials

Supplementary materials can be found at https://www.mdpi.com/1422-0067/21/14/4819/s1.

## Abbreviations

ABA	Abscisic acid
ACC	1-Aminocyclopropane-1-carboxylic acid
ABC	ATP-binding cassette transporter
AGP	Arabinogalactan protein
ARFs	Auxin response factors
Aux/IAA	Auxin/Indole-3 acetic acid
AZ	Abscission zone
BR	Brassinosteroid
BRI1	BR insensitive 1
BAK1	BRI1-associated kinase1
BIN2	BR insensitive 2
BRZ1	Brassinazole resistant 1
cDNA	Complementary deoxyribonucleic acid
CK	Cytokinin
DPA	Days post-anthesis
ECM	Equivalent to the cell wall or apoplast
ER	Endoplasmic reticulum
ERF	Ethylene response factor
ET	ethylene
EXP	Expansin
EXT	Extensin
GA	Gibberellin
GID1	Gibberellin-insensitive dwarf1
IAA	Indole-3-acetic acid
JA	Jasmonic acid
PA	Polyamine
PG	Polygalacturonase
PM	Plasma membrane
PME	Pectin methylesterase
qRT-PCR	Quantitative real-time polymerase chain reaction
RPKM	Reads per kilobase of exon per million mapped reads
SA	Salicylic acid
SAUR	Small auxin up RNA
TGN/EE	The trans-Golgi network and early endosomal compartments
TIR1	Transport inhibitor response 1
XTH	Xyloglucanendotransglucosylase/endohydrolase
