# Supplementary material for: Transcriptome and Hormone Analyses Revealed Insights into Hormonal and Vesicle Trafficking Regulation among Olea europaea Fruit Tissues in Late Development
Source: Int J Mol Sci. 2020 Jul 8;21(14):4819. doi: 10.3390/ijms21144819 (PMC7404322; doi:10.3390/ijms21144819)
Supplement: Supplementary file 1 [file ijms-21-04819-s001.pdf]

Transcriptome and hormone analyses revealed insights into hormonal and vesicle trafficking regulation between *Olea europaea* fruit tissues in late development

Beatriz Briegas[1], Jorge Corbacho[1], Maria C. Parra-Lobato[1], Miguel A. Paredes[1], Juana Labrador[1], Mercedes Gallardo[2] and Maria C. Gomez-Jimenez[1*]

[1]University of Extremadura, Plant Physiology, Avda de Elvas s/n, 06006 Badajoz, Spain. [2]University of Vigo, Plant Physiology, Campus Lagoas-Marcosende, s/n, 36310 Vigo, Spain.
*Correspondence: mcgomez@unex.es

SUPPLEMENTARY MATERIAL

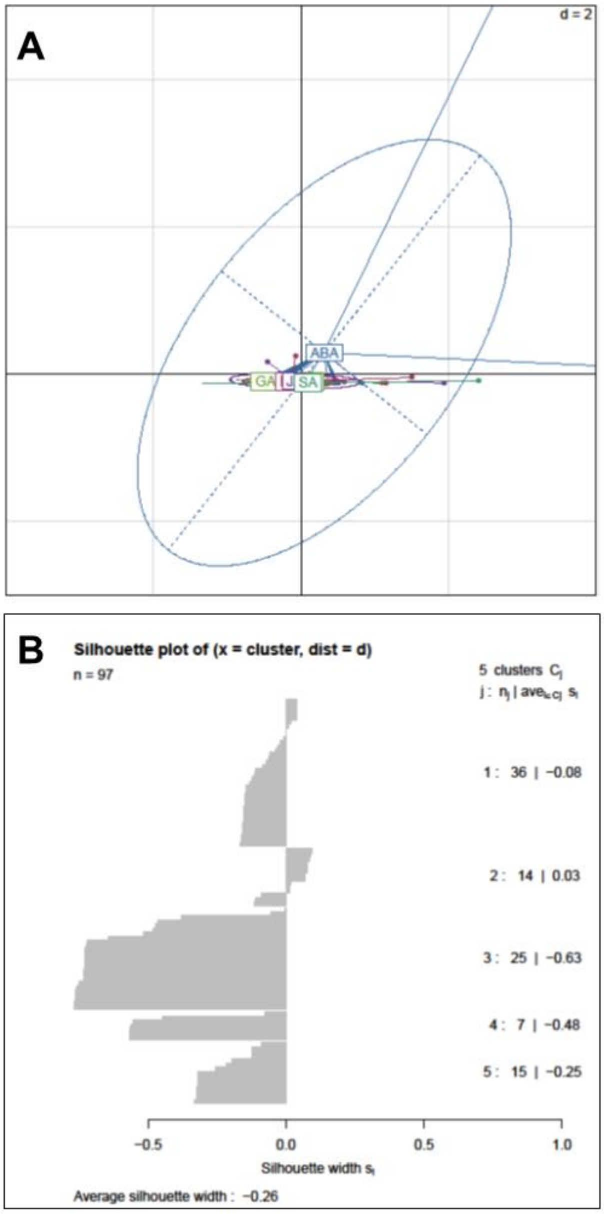

Figure S1. (A) Hierarchical Clustering Analysis (HCA) of differentially expressed genes related to hormone metabolism and signaling in the indicated groups (hormone levels) in olive AZ and fruit at the last fruit ripening. **(B)** Silhouette plot for gene-hormone relationships in AZ and fruit at last stage of fruit ripening.

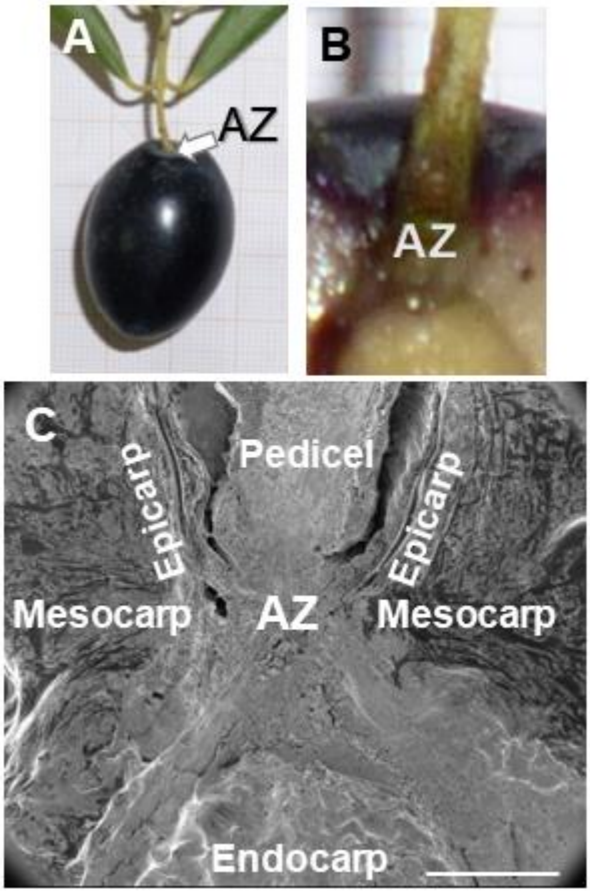

Figure S2. Tissues of olive (*Olea europaea* L. cv Picual) used for this study: AZ and pericarp (epicarp and mesocarp) of fruit at the last stage of ripening. (A) Image of olive ripe-fruit illustrating the location of the AZ between the peduncle (top) and fruit (bottom). (B) Longitudinal section of the transition zone between the peduncle (top) and fruit (bottom) showing the AZ of olive ripe-fruit. (C) Scanning electron micrograph of a longitudinal section of Picual fruit illustrating the position of the tissue samples used for this study: the fruit (epicarp and mesocarp) and their AZ at 217 DPA. Scale bar 1 mm.

Table S1. Fruit- or AZ-enriched genes encoding various cell-wall proteins at the last stage of olive fruit ripening. Sequences were selected after establishing a P<0.01. The table shows the total read count in RPKMx1000 for each gene after normalization across the two samples: fruit-pericarp and their AZ at 217 DPA.

	Uniprot ID	AZ	Fruit	P value	Description
**α-galactosidase**					
	Enriched in fruit				
	Q5DUH8	2.00	26.19	2,27E-07	Alpha galactosidase, gal1 = *Coffea arabica*
**α-1,4-glucan-protein synthase**					
	Enriched in fruit				
	Q2HV87	0.00	150.81	2,70E-44	Alpha-1,4-glucan-protein synthase (UDP-forming) = *Medicago truncatula*
	Enriched in AZ				
	B9RAC8	83.00	54.32	2,41E-03	Alpha-1,4-glucan-protein synthase [UDP-forming] = *Ricinus communis*
**α-glucosidase**					
	Enriched in fruit				
	B9STU2	0.00	3.97	9,77E-04	Neutral alpha-glucosidase ab = *Ricinus communis*
	Enriched in AZ				
	Q9LEC9	12.00	0.00	2,91E-11	Alpha-glucosidase, mal2 = *Solanum tuberosum*
**arabinogalactan protein**					
	Enriched in AZ				
	Q8LCE4	22.56	0.00	1,95E-03	Classical arabinogalactan protein 5, AGP5, At1g35230
**β-1,3-glucanase**					
	Enriched in fruit				
	Q9FXL4	0.00	642.39	8,53E-252	Elicitor inducible beta-1,3-glucanase NtEIG-E76 = *Nicotiana tabacum*
	Enriched in AZ				
	Q68V46	1350.00	0.00	0,00E+00	Beta-1,3-glucanase, glu-4 = *Olea europaea*
	B9RSS2	42.00	0.00	9,31E-10	Endo-1,3-1,4-beta-d-glucanase = *Ricinus communis*
	B9SCU1	94.00	11.13	5,10E-22	Glucan endo-1,3-beta-glucosidase = *Ricinus communis*
**β-galactosidase**					
	Enriched in fruit				
	O81100	0.00	27.16	6,94E-18	Beta-galactosidase, TBG4 = *Solanum lycopersicum*
	Enriched in AZ				
	O65736	25.00	0.00	1,39E-17	Beta-galactosidase = *Cicer arietinum*
	B9N0S6	3.00	0.00	7,81E-03	Beta-galactosidase = *Populus trichocarpa*
**β-glucosidase**					
	Enriched in fruit				
	Q8GVD0	215.00	5033.87	0,00E+00	Beta-glucosidase, bglc = *Olea europaea*
	Enriched in AZ				
	O82151	59.00	0.00	4,81E-35	Beta-D-glucan exohydrolase = *Nicotiana tabacum*
**β-hexosaminidase**					
	Enriched in fruit				
	D3TI69	0.00	27.24	2,84E-14	Beta-hexosaminidase 1 = *Solanum lycopersicum*
**Cellulase**					
	Enriched in fruit				
	Q9XF22	0.00	6.23	3,91E-03	Cellulase = *Nicotiana alata*
	Enriched in AZ				
	Q43149	20.00	0.00	9,31E-10	Cellulase = *Sambucus nigra*
**cellulose synthase**					

	Enriched in fruit				
	B9IKV7	0.00	46.74	8,35E-43	Cellulose synthase = *Populus trichocarpa*
	B9S9V9	4.00	24.16	1,05E-10	Cellulose synthase = *Ricinus communis*
	D7U7F7	0.00	6.06	4,88E-04	Cellulose synthase = *Vitis vinifera*
	B9GFE1	0.00	12.33	3,64E-12	Cellulose synthase = *Populus trichocarpa*
	D7T308	0.00	11.84	2,33E-10	Cellulose synthase = *Vitis vinifera*
	Enriched in AZ				
	Q6XP47	13.00	0.00	9,09E-13	Cellulose synthase, StCesA1 = *Solanum tuberosum*
	C6KF43	11.00	0.00	2,38E-07	Cellulose synthase catalytic subunit, cesA7 = *Gossypium hirsutum*
	Q4PKB6	5.00	0.00	6,10E-05	Cellulose synthase CesA1 = *Boehmeria nivea*
	Q45KQ0	26.00	0.00	1,73E-18	Cellulose synthase-like protein CslE = *Nicotiana tabacum*
	Q3Y6V1	14.00	0.00	4,66E-10	Cellulose synthase-like protein CslG = *Nicotiana tabacum*
	B9I7Q4	12.00	0.00	9,09E-13	Cellulose synthase = *Populus trichocarpa*
	B9RYN4	3.00	0.00	7,81E-03	Cellulose synthase = *Ricinus communis*
	D7T7B9	6.00	0.00	7,81E-03	Cellulose synthase = *Vitis vinifera*
	D7THX8	11.00	0.00	1,14E-13	Cellulose synthase = *Vitis vinifera*
	D7SUS1	5.00	0.00	1,22E-04	Cellulose synthase = *Vitis vinifera*
**chitinase**					
	Enriched in fruit				
	A5B1C7	25.00	71.65	3,67E-05	Chitinase = *Vitis vinifera*
	D7T2C4	19.00	113.28	1,81E-14	Chitinase = *Vitis vinifera*
	Enriched in AZ				
	Q9FS45	134.00	10.25	5,46E-30	Chitinase = *Vitis vinifera*
	A1IJ67	10.00	0.00	3,91E-03	Class IV chitinase = *Nicotiana tabacum*
	D7SSZ8	350.00	0.00	1,92E-93	Chitinase = *Vitis vinifera*
**endo-1,4-β-glucanase**					
	Enriched in fruit				
	Q9ZSP9	0.00	20.80	7,28E-12	Endo-beta-1,4-D-glucanase, Cel8 = *Solanum lycopersicum*
	B9S075	103.00	146.00	4,49E-03	Endo-1,4-beta-glucanase = *Ricinus communis*
	Enriched in AZ				
	B9RLZ9	4.00	0.00	7,81E-03	Endo-1,4-beta-glucanase = *Ricinus communis*
	Q0KIX2	68.00	0.00	3,67E-40	Endoglucanase, CmEGase1 = *Cucumis melo*
**endo-β-mannanase**					
	Enriched in AZ				
	C7A7X6	44.00	18.47	8,72E-05	Endo-beta-mannanase, MAN1 = *Actinidia arguta*
**expansin**					
	Enriched in fruit				
	Q9ZP37	0.00	232.00	7,35E-51	Alpha-expansin, Nt-EXPA3 = *Nicotiana tabacum*
	B9R8E5	0.00	457.03	1,31E-98	Alpha-expansin 11 = *Ricinus communis*
	Q9M5I7	132.00	658.56	4,56E-56	Alpha-expansin 3 = *Triphysaria versicolor*
	B9S4E4	0.00	2111.11	0,00E+00	Alpha-expansin 8 = *Ricinus communis*
	Q84UT0	0.00	247.96	4,70E-52	Expansin, Vexp-1 = *Vitis vinifera*
	B7U8J4	52.00	1418.63	1,22E-244	Expansin, CDK3 = *Diospyros kaki*
	A5BA94	0.00	120.83	5,17E-26	Expansin = *Vitis vinifera*
	Enriched in AZ				
	B9IGR9	10.00	0.00	3,91E-03	PtEXPA13 = *Populus trichocarpa*
**extensin**					
	Enriched in fruit				
	Q39600	0.00	216.58	7,98E-40	Extensin, cyc17 = *Catharanthus roseus*

	P13983	0.00	213.97	2,39E-112	Extensin, HRGPNT3 = *Nicotiana tabacum*
	B9RPC0	9.29	41.51	3,64E-08	leucine-rich repeat family protein / extensin family protein LRX1 = *Ricinus communis*
	Enriched in AZ				
	C3VPW8	9.00	0.00	7,81E-03	Extensin = *Lithospermum erythrorhizon*
**glycosyl hydrolase**					
	Enriched in fruit				
	D7U290	0.00	2.71	7,81E-03	Glycosyl hydrolase 1 family protein = *Vitis vinifera*
	Enriched in AZ				
	B9HAA3	32.00	0.00	1,16E-10	Glycosyl hydrolase 17 family = *Populus trichocarpa*
	D7TQ09	19.00	4.08	1,17E-04	Glycosyl hydrolase 17 family = *Populus trichocarpa*
	B9GQH4	10.00	0.00	1,95E-03	Glycosyl hydrolase 18 family protein = *Populus trichocarpa*
	B9HYK4	404.00	39.21	8,46E-70	Glycosyl hydrolase 18 family protein = *Populus trichocarpa*
	A5BD25	4.00	0.00	7,81E-03	Glycosyl hydrolase 18 family protein = *Vitis vinifera*
	D7U285	120.00	58.68	2,47E-11	Glycosyl hydrolase 1 family protein = *Vitis vinifera*
	D7U288	8.00	0.00	9,54E-07	Glycosyl hydrolase 1 family protein = *Vitis vinifera*
**laccase**					
	Enriched in AZ				
	B2M153	518.00	0.00	4,51E-277	Laccase = *Rosa hybrid cultivar*
	B9H7Z9	170.00	35.39	9,82E-42	Laccase = *Populus trichocarpa*
	B9IEA5	14.00	0.00	2,98E-08	Laccase = *Populus trichocarpa*
**lyase**					
	Enriched in AZ				
	B9SMY4	31.00	0.00	8,67E-19	Lyase = *Ricinus communis*
	B9SMM6	156.00	0.00	4,91E-91	Lyase = *Ricinus communis*
	B9SML0	215.00	0.00	6,88E-136	Lyase = *Ricinus communis*
**pectin methylesterase/ Pectinesterase**					
	Enriched in fruit				
	Q84V57	0.00	368.45	1,16E-180	Pectinesterase = *Nicotiana benthamiana*
	B9H3W4	0.00	9.49	3,91E-03	Pectinesterase = *Populus trichocarpa*
	D7SXF6	0.00	8.43	7,81E-03	Pectinesterase = *Vitis vinifera*
	P09607	0.00	24.84	1,82E-12	Pectinesterase 2.1 (PE 2.1) (Pectin methylesterase 2.1), PME2.1 = *Solanum lycopersicum*
	Enriched in AZ				
	A0ZNK0	10.00	0.00	7,63E-06	Pectin methylesterase 2, PcPME2 = *Pyrus communis*
	B9SP63	4.00	0.00	7,81E-03	Pectinesterase = *Ricinus communis*
	B9RD90	8.00	0.00	2,44E-04	Pectinesterase = *Ricinus communis*
**polygalacturonase**					
	Enriched in fruit				
	D7U2D3	2.00	67.78	2,38E-23	Polygalacturonase = *Vitis vinifera*
	D7U2D3	2.00	67.78	2,38E-23	Polygalacturonase = *Vitis vinifera*
	Enriched in AZ				
	B9RCG6	24.00	0.00	2,91E-11	Polygalacturonase = *Ricinus communis*
	B1PK08	616.00	114.23	3,23E-100	Polygalacturonase = *Olea europaea*
	A7PZL3	10.00	0.00	3,05E-05	Polygalacturonase = *Vitis vinifera*
**ramnose synthase**					
	Enriched in AZ				
	Q9SYM5	206.78	84.21	6,03E-30	Probable rhamnose biosynthetic enzyme, RHM1, At1g78570
**xyloglucan endotransglucosylase/hydrolase**					
	Enriched in AZ				
	Q6EJD2	72.00	0.00	2,17E-19	Xyloglucan endotransglucosylase, XTH-1 = *Beta vulgaris*

C0IRH3	105.00	38.01	5,79E-10	Xyloglucan endotransglucosylase/hydrolase 14 = *Actinidia deliciosa*
C0IRG4	80.00	0.00	2,12E-22	Xyloglucan endotransglucosylase/hydrolase 5 = *Actinidia deliciosa*
**xyloglucan:xyloglucosyl transferase**				
Enriched in AZ				
B9HAM2	25.00	0.00	9,54E-07	Xyloglucan:xyloglucosyl transferase = *Populus trichocarpa*
A5BND5	94.00	0.00	2,58E-26	Xyloglucan:xyloglucosyl transferase = *Vitis vinifera*

Table S2. Fruit- or AZ-enriched genes encoding various plant-hormone metabolism and signaling proteins at the last stage of olive fruit ripening. Sequences were selected after establishing a P<0.01. The table shows the total read count in RPKMx1000 for each gene after normalization across the two samples: fruit-pericarp and their AZ at 217 DPA.

	Uniprot ID	AZ	Fruit	P value	Description
Ethylene					
	Enriched in fruit				
	C6KMJ4	1423.76	4795.00	0.00E+00	ACC oxidase, ACO1 = *Boea hygrometrica*
	A1E4D3	13.51	611.00	0.00E+00	Ethylene receptor, ETR1 = *Coffea canephora*
	Q2PQJ0	0.00	68.00	2.46E-38	EIN3-binding F-box protein 1 (EIN3-binding F-box protein 2), EBF2 EBF1 = *Solanum lycopersicum*
	Q39026	0.00	45.00	4.44E-16	Mitogen-activated protein kinase 6 (AtMPK6), MPK6 At2g43790 F18O19.10
	O48631	0.00	29.00	1.86E-09	Ethylene-forming-enzyme-like dioxygenase = *Prunus armeniaca*
	Q8GTL5	0.00	10.00	2.44E-04	S-adenosylmethionine synthase (AdoMet synthase) (Methionine adenosyltransferase) (MAT), SAMS = *Carica papaya*
	Q9SXS8	100.74	169.00	4.60E-03	Ethylene-responsive transcription factor 3 (Ethylene-responsive element-binding factor 3 homolog) (Ethylene-responsive element-binding factor 5) (EREBP-5) (NtERF5), ERF3 ERF-5 ERF5 = *Nicotiana tabacum*
	Enriched in AZ				
	Q5IWL7	319.17	0.00	1.73E-77	Ethylene-responsive element binding protein 5 = *Nicotiana tabacum*
	Q84QD4	166.12	18.00	3.87E-62	EIL1 = *Nicotiana tabacum*
	A7Q0V4	326.51	70.00	3.00E-53	S-adenosylmethionine synthase 5 (AdoMet synthase 5) (Methionine adenosyltransferase 5) (MAT 5), METK5= *Vitis vinifera*
	Q9SFB0	91.25	0.00	2.80E-45	MATE efflux family protein FRD3 (MATE citrate transporter) (Protein DTX43) (Protein FERRIC REDUCTASE DEFECTIVE 3) (AtFRD3) (Protein MANGANESE ACCUMULATOR 1), FRD3 DTX43 MAN1 At3g08040 F17A17.38 T8G24.8
	Q2PQJ1	79.01	2.00	6.88E-44	EIN3-binding F-box protein 1 (EIN3-binding F-box protein 2), EBF1 EBF2 = *Solanum lycopersicum*
	Q9FHW7	608.18	207.00	5.50E-29	SKP1-like protein 1B (SKP1-like 2) (UFO-binding protein 2), SKP1B ASK2 UIP2 At5g42190 MJC20.30
	Q38950	256.67	111.00	2.31E-29	Serine/threonine-protein phosphatase 2A 65 kDa regulatory subunit A beta isoform (AtA beta) (PP2A. subunit A. beta isoform), PP2AA2 DF1 At3g25800 K13N2.14
	Q6R567	133.59	11.00	1.49E-23	E3 ubiquitin-protein ligase RMA1H1 (EC 6.3.2.-) (Protein RING membrane-anchor 1 homolog 1), RMA1H1 = *Capsicum annuum*
	P31237	66.87	0.00	1.36E-20	1-aminocyclopropane-1-carboxylate oxidase (ACC oxidase), ACO = *Actinidia deliciosa*
	A9P822	242.34	105.00	3.08E-19	S-adenosylmethionine synthase 1 (AdoMet synthase 1) (Methionine adenosyltransferase 1) (MAT 1), METK1 = *Populus trichocarpa*
	Q9LW49	83.70	0.00	3.47E-18	Ethylene-responsive transcription factor 4 (Ethylene-responsive element-binding factor 3) (EREBP-3) (Ethylene-responsive element-binding factor 4 homolog) (NsERF3), ERF4 ERF-3 ERF3 = *Nicotiana sylvestris*

	Q32W74	56.76	0.00	3.55E-15	Ethylene responsive element binding protein C2 = *Capsicum annuum*
	Q9SJL8	343.58	215.00	1.59E-12	S-adenosylmethionine synthase 3 (AdoMet synthase 3) (Methionine adenosyltransferase 3) (MAT 3), METK3 At2g36880 T1J8.6
	B9HK87	15.47	0.00	3.73E-09	Ethylene-insensitive 3d, EIN3D = *Populus trichocarpa*
	D8VD38	35.15	0.00	1.86E-09	Ethylene response factor 11, ERF11 = *Actinidia deliciosa*
	B9S1K1	35.26	7.00	3.54E-09	S-adenosylmethionine-dependent methyltransferase = *Ricinus communis*
	Q9XET8	9.63	0.00	2.38E-07	Ethylene receptor (Ethylene receptor homolog) ETR4 = *Solanum lycopersicum*
	Q39227	21.23	0.00	1.19E-07	24-methylenesterol C-methyltransferase 2 (24-sterol C-methyltransferase 2) (Sterol-C-methyltransferase 2) (EC 2.1.1.143) (Protein COTYLEDON VASCULAR PATTERN 1), SMT2 CVP1 At1g20330 F14O10.7
	B3FIB0	13.30	0.00	1.91E-06	Ethylene receptor ETR2 = *Actinidia deliciosa*
	Q6V397	4.58	0.00	3.81E-06	EIN2 = *Petunia hybrida*
	O22587	6.99	0.00	1.53E-05	Ethylene receptor homolog, NTHK1 = *Nicotiana tabacum*
	A6ZI64	110.63	52.00	1.96E-05	Putative ethylene-responsive element binding protein = *Salvia miltiorrhiza*
	P28186	310.18	231.00	4.28E-04	Ras-related protein ARA-3, ARA-3 At3g46060 F12M12_30
	Q6RJ36	14.10	0.00	4.88E-04	Ethylene responsive factor 2 (Ethylene-binding protein), ERF2 = *Solanum lycopersicum*
	Q9SHE7	115.38	53.00	4.52E-04	Ubiquitin-NEDD8-like protein RUB1 [Cleaved into: Ubiquitin; NEDD8-like protein RUB1 (Ubiquitin-related protein 1) (AtRUB1)], RUB1 NEDD8 UBQ15 At1g31340 T19E23.13
	Q8W231	7.05	0.00	4.88E-04	Putative serine/threonine-specific protein kinase, CTR1 = *Pyrus communis*
	Q6V398	5.16	0.00	9.77E-04	EIL1 = *Petunia hybrida*
	O22174	17.04	0.00	1.95E-03	Ethylene-responsive transcription factor ERF008, ERF008 At2g23340 T20D16.3
Auxin					
	Enriched in fruit				
	Q940X7	172.31	1565.00	3.56E-91	RING-box protein 1a (At-Rbx1;1) (Protein RING of cullins 1) (RBX1-2) (RBX1a-At), RBX1A ROC1 At5g20570 F7C8.160
	P31414	0.00	16.00	2.91E-11	Pyrophosphate-energized vacuolar membrane proton pump 1 (Pyrophosphate-energized inorganic pyrophosphatase 1) (H(+)-PPase 1) (Vacuolar proton pyrophosphatase 1) (Vacuolar proton pyrophosphatase 3) = AVP1 AVP AVP-3 AVP3 At1g15690 F7H2.3
	B9RUW0	0.00	51.00	2.33E-10	Auxin-responsive protein IAA1 = *Ricinus communis*
	Q5ZF70	0.00	35.00	1.19E-07	Auxin resistance protein, axr2 = *Plantago major*
	D1MWZ6	69.44	228.00	4.33E-07	Auxin-repressed protein, CitAuR = *Citrullus lanatus*
	B9H0Z6	0.00	64.00	1.91E-06	SAUR family protein, SAUR29 = *Populus trichocarpa*
	D7M6D2	0.00	12.00	6.10E-05	Auxin efflux carrier family protein, ARALYDRAFT_486870
	Q9LTX2	0.00	6.00	9.77E-04	Transport inhibitor response 1-like protein (TIR1-like protein), At5g49980 K9P8.12
	Q9XEY3	18.15	54.00	3.66E-03	Nt-iaa4.3 deduced protein = *Nicotiana tabacum*
	Enriched in AZ				
	O04951	277.95	0.00	3.37E-80	Serine/threonine-protein phosphatase PP2A-5 catalytic subunit (Protein phosphatase 2A isoform 5), PP2A5 At1g69960 F20P5.30 T17F3.1
	B9HCL2	85.41	0.00	1.18E-38	Auxin influx carrier component, PtrAUX1 = *Populus trichocarpa*
	D9ZIM5	148.34	15.00	5.73E-35	ARF domain class transcription factor, IAA1 = Malus domestica
	B9H216	58.72	0.00	2.47E-32	F-box family protein, FBL5 = *Populus trichocarpa*
	Q9FHW7	608.18	207.00	5.50E-29	SKP1-like protein 1B (SKP1-like 2) (UFO-binding protein 2), SKP1B ASK2 UIP2 At5g42190 MJC20.30
	P46423	196.54	20.00	6.29E-26	Glutathione S-transferase (EC 2.5.1.18) (25 kDa auxin-binding protein) (GST class-phi) = *Hyoscyamus muticus*
	A5C819	39.24	0.00	2.65E-23	Putative uncharacterized protein = *Vitis vinifera*
	B9IGX9	82.93	19.00	4.48E-15	Auxin efflux carrier component. auxin transport protein = *Populus trichocarpa*
	Q1W389	52.41	0.00	9.09E-13	Auxin-regulated protein = *Striga asiatica*
	C0SU68	25.91	0.00	3.64E-12	Auxin influx carrier protein, ZeLAX1 = *Zinnia elegans*

	B9I233	118.51	7.00	5.52E-12	SAUR family protein, SAUR22 = *Populus trichocarpa*
	C0SU69	23.35	0.00	5.82E-11	Auxin influx carrier protein, ZeLAX2 = *Zinnia elegans*
	B9SHS8	27.30	0.00	2.91E-11	Auxin-induced protein 5NG4 = *Ricinus communis*
	Q949J8	21.61	0.00	4.66E-10	Putative auxin growth promotor protein = *Solanum lycopersicum*
	B9R824	27.77	0.00	1.16E-10	Auxin-induced protein 5NG4 = *Ricinus communis*
	C8CBW3	30.78	0.00	1.19E-07	Auxin/indole-3-acetic acid 3, IAA3 = *Solanum tuberosum*
	B9RQI4	16.08	0.00	3.81E-06	Auxin-responsive protein IAA27 = *Nicotiana tabacum*
	Q2LAJ3	10.63	1.00	4.65E-06	Auxin response factor 2, ARF2 = *Solanum lycopersicum*
	B9SN97	10.60	0.00	1.91E-06	Indole-3-acetic acid-amido synthetase GH3.3 = *Ricinus communis*
	P40691	15.20	0.00	6.10E-05	Auxin-induced protein PCNT115 = *Nicotiana tabacum*
	B9RJT7	5.24	0.00	9.77E-04	TRANSPORT INHIBITOR RESPONSE 1 protein = *Ricinus communis*
	Q9SHE7	115.38	53.00	4.52E-04	Ubiquitin-NEDD8-like protein RUB1 [Cleaved into: Ubiquitin; NEDD8-like protein RUB1 (Ubiquitin-related protein 1) (AtRUB1)], RUB1 NEDD8 UBQ15 At1g31340 T19E23.13
	D7SH69	9.66	3.00	1.32E-03	Whole genome shotgun sequence of line PN40024. Scaffold 0.assembly12x (Fragment) = *Vitis vinifera*
	B9S0L2	3.79	0.00	3.91E-03	Auxin response factor = *Ricinus communis*
	B9S1E4	2.70	0.00	1.95E-03	Auxin response factor = *Ricinus communis*
	D9IA29	2.69	0.00	1.95E-03	Auxin response factor 19, ARF19 = *Solanum lycopersicum*
	C7E4R3	4.01	0.00	7.81E-03	Transport inhibitor response 1, TIR1 = *Nicotiana tabacum*
Abscisic acid					
	Enriched in fruit				
	Q39026	0.00	45.00	4.44E-16	Mitogen-activated protein kinase 6 (AtMPK6) (MAP kinase 6), MPK6 At2g43790 F18O19.10
	Q944A7	0.00	29.00	2.27E-13	Probable serine/threonine-protein kinase At4g35230
	P43291	0.00	39.00	9.09E-13	Serine/threonine-protein kinase SRK2A (EC 2.7.11.1) (Arabidopsis protein SK1) (OST1-kinase-like 7) (SNF1-related kinase 2.4) (SnRK2.4), SRK2A ASK1 OSKL7 SNRK2.4 At1g10940 T19D16.14
	Q8RXD3	0.00	13.00	4.88E-04	E3 ubiquitin-protein ligase AIP2 (EC 6.3.2.-) (ABI3-interacting protein 2), AIP2 At5g20910 F22D1.80
	Q8LGH4	0.00	4.00	1.95E-03	Cullin-4 (AtCUL4), CUL4 At5g46210 MDE13.3
	B9SE77	59.10	447.00	4.33E-29	Abscisic stress ripening protein = *Ricinus communis*
	A0SE34	0.00	9.00	3.05E-05	9-cis-epoxycarotenoid dioxygenase 5, NCED5 = *Citrus clementina*
	B9S5U9	0.00	5.00	7.81E-03	Protein phosphatase 2c = *Ricinus communis*
	Q9MAM1	0.00	6.00	7.81E-03	CBL-interacting serine/threonine-protein kinase 9 (EC 2.7.11.1) (SNF1-related kinase 3.12) (SOS2-like protein kinase PKS6), CIPK9 PKS6 SnRK3.12 At1g01140 F6F3.28 T25K16.13
	Enriched in AZ				
	B9RNU7	1060.01	145.00	1.01E-205	Protein phosphatase 2c = *Ricinus communis*
	Q2TUW1	1196.07	20.74	0.00E+00	Abscisic stress ripening-like protein = *Glycine max*
	Q9ZUU4	235.29	5.00	1.24E-58	Ribonucleoprotein At2g37220. Chloroplastic, At2g37220 F3G5.1
	O80653	11.96	0.00	2.38E-07	At1g77180/T14N5_5 (Putative nuclear protein) (Putative uncharacterized protein At1g77180), At1g77180 T14N5.5
	P22240	74.35	0.00	1.86E-09	Abscisic acid and environmental stress-inducible protein TAS14 (Dehydrin TAS14) = *Solanum lycopersicum*
	A9QNE7	9.10	0.00	1.22E-04	ABA 8'-hydroxylase, CYP707A1 = *Solanum lycopersicum*
	B9SVA1	93.10	13.00	6.79E-25	Dihydrolipoamide succinyltransferase component of 2-oxoglutarate dehydrogenase = *Ricinus communis*
	Q9M3V1	6.45	0.00	3.91E-03	Protein phpsphatase 2C (PP2C), pp2C1 = Fagus sylvatica
	B9RK51	7.44	0.00	2.44E-04	Protein phosphatase 2c = *Ricinus communis*
	B9RVV7	18.84	0.00	1.53E-05	Protein phosphatase 2c = *Ricinus communis*
	B9R8Q9	22.39	0.00	1.49E-08	Protein phosphatase 2c = *Ricinus communis*

	C8KHU4	36.94	0.00	1.42E-14	Protein phosphatase 2C AHG3 homolog, SlPP2C-2 = *Solanum lycopersicum*
	B9RJK5	94.22	0.00	2.07E-25	Protein phosphatase 2c putative = *Ricinus communis*
	Q5PNS9	120.83	0.00	1.40E-45	Probable protein phosphatase 2C 64 (AtPP2C64) (EC 3.1.3.16)
	Q9FKX4	129.00	0.00	8.76E-47	Probable protein phosphatase 2C 79 (AtPP2C79), At5g66080 K2A18.16
	C8KHU3	93.75	0.00	5.47E-48	Protein phosphatase 2C ABI2 homolog, SlPP2C-1 = *Solanum lycopersicum*
Jasmonate					
	Enriched in fruit				
	B9SG47	0.00	43.00	5,82E-11	Sigma factor sigb regulation protein rsbq (methyl esterase activity, MES: methyl jasmonate (MeJA) esterase activity , methyl salicylate (MeSA) esterase activity and methyl indole-3-acetate (MeIAA) esterase activity)
	O24370	0	161.29	4,16E-123	Lipoxygenase = *Solanum tuberosum*
	Q39026	0.00	45.00	4.44E-16	Mitogen-activated protein kinase 6 (AtMPK6) (MAP kinase 6), MPK6 At2g43790 F18O19.10
	Enriched in AZ				
	B9SEM5	12.91	0.00	9,77E-04	Acyl-protein thioesterase (methyl indole-3-acetate esterase activity; methyl jasmonate esterase activity; methyl salicylate esterase activity) = *Ricinus communis*
	Q8S8S9	16.47	0.00	1,22E-04	At2g23620 (Putative acetone-cyanohydrin lyase), methyl indole-3-acetate esterase activity; methyl jasmonate esterase activity; methyl salicylate esterase activity; systemic acquired resistance
	Q8LAH7	341.39	0.00	4.96E-119	12-oxophytodienoate reductase 1 (12-oxophytodienoate-10.11-reductase 1) (AtOPR1) (OPDA-reductase 1) (FS-AT-I), OPR1 At1g76680 F28O16.5
	B9RRB8	83.54	5.00	1,38E-24	12-oxophytodienoate reductase opr = *Ricinus communis*
	B6D1W5	44.75	16.00	5,70E-11	Lipoxygenase = *Olea europaea*
	O24371	18.23	0.00	4,44E-16	Lipoxygenase =*Solanum tuberosum*
	Q9M464	13.10	0.00	4,77E-07	Allene oxide synthase AOS = *Solanum lycopersicum*
	Q9SQK8	22.49	0.00	2.38E-07	Jasmonic acid 3, LEJA3 = *Solanum lycopersicum*
	D8V3L7	112.67	7.00	5.01E-24	Plastid jasmonates ZIM-domain protein = *Hevea brasiliensis*
	A7XXZ0	26.45	0.00	9.54E-07	Jasmonate ZIM-domain protein = *Solanum lycopersicum*
	B0VXR3	21.26	0.00	3.64E-12	JAR1-like protein, JAR6 = *Nicotiana attenuata*
	A6NAB4	4.93	0.00	1.95E-03	Myc2 bHLH protein = *Vitis vinifera*
Cytokinins					
	Enriched in fruit				
	D7TDN8	0.00	5.00	1.95E-03	cytokinin dehydrogenase = *Vitis vinifera*
	Enriched in AZ				
	Q3ECF7	8.05	0.00	4.88E-04	Uncharacterized protein, At1g69040
Polyamine					
	Enriched in fruit				
	Q8GTQ5	0.00	12.00	4.88E-04	Spermidine synthase, MdSPDS2a = *Malus domestica*
	Enriched in AZ				
	B9SIY7	142.06	0.00	5.47E-48	S-adenosylmethionine decarboxylase proenzyme = *Ricinus communis*
	A5AFT0	133.33	0.00	2.80E-45	S-adenosylmethionine decarboxylase proenzyme = *Vitis vinifera*
	B9RT51	20.33	4.00	1.38E-06	Protein arginine n-methyltransferase = *Ricinus communis*
	B3Y023	337.01	6.00	1.24E-210	Arginine decarboxylase, PpADC = *Prunus persica*
	D2K8S6	52.42	0.00	5.55E-17	Spermidine synthase = *Olea europaea*
Brassinosteroid					

	Enriched in fruit				
	B9RI66	0.00	51.00	3.61E-34	BRASSINOSTEROID INSENSITIVE 1-associated receptor kinase 1 = *Ricinus communis*
	Q944A7	0.00	29.00	2.27E-13	Probable serine/threonine-protein kinase At4g35230
	Enriched in AZ				
	Q39011	252.63	75.00	7.52E-31	Shaggy-related protein kinase eta (ASK-eta) (Protein BRASSINOSTEROID INSENSITIVE 2) (Protein ULTRACURVATA 1), ASK7 BIN2 DWF12 UCU1 At4g18710 F28A21.120
	B9S318	11.61	0.00	9.77E-04	Brassinosteroid-regulated protein BRU1 = *Ricinus communis*
	B9RAQ8	5.79	0.00	4.88E-04	BRASSINOSTEROID INSENSITIVE 1-associated receptor kinase 1 = *Ricinus communis*
Salicylic acid					
	Enriched in AZ				
	Q8H6W0	35.33	3.00	2.44E-17	Phenylalanine ammonia-lyase, PAL1 = *Coffea canephora*
	O23924	7.01	0.00	3.05E-05	Phenylalanine ammonia-lyase = *Digitalis lanata*
	Q6RYA0	584.61	51.00	1.32E-98	Salicylic acid-binding protein 2 = *Nicotiana tabacum*
	Q9FGY9	3.69	0.00	3.91E-03	Peptide-N(4)-(N-acetyl-beta-glucosaminyl)asparagine amidase (Peptide:N-glycanase) (AtPNG1), PNG1 At5g49570 K6M13.12
	Q8S8S9	16.47	0.00	1.22E-04	At2g23620 (Putative acetone-cyanohydrin lyase) = At2g23620
	B9H966	12.47	0.00	3.81E-06	BOP/NPR1/NIM1-like regulatory protein = *Populus trichocarpa*
	B9S3I0	12.42	0.00	2.38E-07	Regulatory protein NPR1 = *Ricinus communis*
Gibberellin					
	Enriched in fruit				
	Q9SLQ9	0.00	139.00	1.06E-43	Gibberellin 3beta-hydroxylase, Nty = *Nicotiana tabacum*
	B9SME4	0.00	77.00	1.29E-26	Gibberellin 20-oxidase = *Ricinus communis*
	Q42698	0.00	58.00	8.67E-19	Geranylgeranyl pyrophosphate synthase. chloroplastic (GGPP synthase) (GGPS) ((2E.6E)-farnesyl diphosphate synthase) (Dimethylallyltranstransferase) (Farnesyl diphosphate synthase) (Farnesyltranstransferase) (Geranyltranstransferase), GGPS1 GGC1 = *Catharanthus roseus*
	Q1ZYL0	0.00	19.00	2.98E-08	Geranylgeranyl reductase = *Olea europaea*
	Enriched in AZ				
	Q8S4W7	35.59	19.00	4.90E-04	DELLA protein GAI1 (Gibberellic acid-insensitive mutant protein 1) (VvGAI1), GAI1 = *Vitis vinifera*
	B9SST2	9.29	0.00	3.05E-05	Chitin-inducible gibberellin-responsive protein = *Ricinus communis*
	C6GMF4	18.07	0.00	3.81E-06	Gibberellin 2-oxidase, gao = *Nicotiana plumbaginifolia*
	A4URE9	27.21	0.00	3.73E-09	Gibberellin 2-oxidase 5, GA2ox5 = *Nicotiana tabacum*
	B9S2K0	92.06	0.00	1.86E-09	Gibberellin-regulated protein 3 = *Ricinus communis*
	B9S2N0	17.29	0.00	1.86E-09	Chitin-inducible gibberellin-responsive protein = *Ricinus communis*
	B9SFJ8	238.64	0.00	3.45E-77	Gibberellin receptor GID1 = *Ricinus communis*
	Q1A7S9	12.85	3.00	7.54E-03	Geranylgeranyl pyrophosphate synthase 2, GGPS2 = *Solanum lycopersicum*
	B9SVW8	12.78	0.00	1.53E-05	Geranylgeranyl pyrophosphate synthase = *Ricinus communis*
	Q9SFB0	91.25	0.00	2.80E-45	MATE efflux family protein FRD3 (MATE citrate transporter) (Protein DTX43) (Protein FERRIC REDUCTASE DEFECTIVE 3) (AtFRD3) (Protein MANGANESE ACCUMULATOR 1), FRD3 DTX43 MAN1 At3g08040 F17A17.38 T8G24.8

Table S3. Fruit- or AZ-enriched genes encoding various vesicle trafficking proteins at the last stage of olive fruit ripening. Sequences were selected after establishing a P<0.01. The table shows the total read count in RPKMx1000 for each gene after normalization across the two samples: fruit-pericarp and their AZ at 217 DPA.

	Uniprot ID	ZA	Fruit	P value	Putative ortholog	Description
**Tubulin family**						
	Enriched in fruit					
	D7TVZ8	0.00	203.53	$6.32E^{-59}$	AT5G12250.1, TUB6	Tubulin = *Vitis vinifera*
	Q3EA24	0.00	41.37	$4.44E^{-16}$	AT4G14960.1, TUA6	AT4G14960 protein
	B9S382	22.01	949.23	$0.00E^{+00}$	AT5G23860.2, TUB8	Tubulin beta chain = *Ricinus communis*
	P29512	180.74	1812.59	$0.00E^{+00}$	AT5G62700.1, TUB3	Tubulin beta-2/beta-3 chain. TUBB2 TUB2 At5g62690 MRG21.11; TUBB3 TUB3 At5g62700 MRG21.12
	Enriched in AZ					
	E0CNV2	6.75	0.00	$3.91E^{-03}$	AT1G50010.1, TUA2	Tubulin = *Vitis vinifera*
	B9SB77	41.06	0.00	$2.22E^{-16}$	AT5G12250.1, TUB6	Tubulin beta chain = *Ricinus communis*
**Actin family**						
	Enriched in fruit					
	B9SXZ4	0.00	47.22	$1.16E^{-10}$	AT3G53750.1, ACT3	Actin = Ricinus communis
	P53496	0.00	7.95	$3.91E^{-03}$	AT3G12110.1, ACT11	Actin-11. ACT11 At3g12110 T21B14.7 T23B7.5 T21B14_108
	D7U423	285.13	834.96	$1.85E^{-64}$	AT5G09810.1, ACT7	Actin = *Vitis vinifera*
	Enriched in AZ					
	D7SMF4	27.92	0.00	$1.16E^{-10}$	AT1G13180.1, DIS1, ARP3, ATARP3	Actin = *Vitis vinifera*
	B9RR79	84.21	0.00	$3.16E^{-30}$	AT5G09810.1, ACT7	Actin = *Ricinus communis*
**Kinesin-like protein family**						
	Enriched in fruit					
	B9RB32	0.00	21.29	$7.11E^{-15}$	AT1G27500.1, KLCR3	Kinesin light chain = *Ricinus communis*
**Small GTPase superfamily. RAB family**						
	Enriched in fruit					
	D7T2X5	0.00	1244.83	$2.22E^{-235}$	AT5G47960.1 ATRABA4D, RABA4D, RAB GTPase homolog	RAB GTPase = *Vitis vinifera*
	A9PCE2	0.00	62.01	$3.64E^{-12}$	AT1G09630.1, ATRAB11C, ATRABA2A, ATRAB-A2A, RAB-A2A,	RAB GTPase = *Populus trichocarpa*
	D7U0K6	0.00	35.17	$9.54E^{-07}$	AT5G45130.1, ATRAB5A, ATRABF2A, RABF2A, RAB5A, RHA1, ATRAB-	RAB GTPase = *Vitis vinifera*
	B9RRP3	0.00	20.74	$1.22E^{-04}$	AT4G19640.1, ARA7, ARA-7, ATRABF2B, ATRAB5B, RABF2B,	RAB GTPase = *Ricinus communis*

D7SJ99	0.00	21.40	$1.22E^{-04}$	AT1G18200.1, AtRABA6b, RABA6b (RabH)	RAB GTPase = *Vitis vinifera*
B9N9L9	0.00	16.89	$9.77E^{-04}$	AT5G60860.1, AtRABA1f, RABA1f, RAB GTPase homolog A1F	RAB GTPase = *Populus trichocarpa*
B9IQR9	13.88	254.62	$4.73E^{-35}$	AT3G15060.1, AtRABA1g, RABA1g,RAB GTPase homolog A1G	RAB GTPase = *Populus trichocarpa*
A5BGY6	4.42	63.42	$4.62E^{-10}$	AT5G65270.1,AtRABA4a, RABA4a,RAB GTPase homolog A4A	RAB GTPase = *Vitis vinifera*
C0LSK7	73.57	724.47	$4.71E^{-164}$	AT3G59920.1, ATGDI2, GDI2, RAB GDP dissociation inhibitor 2	Rab GDP dissociation inhibitor. GDI = *Nicotiana benthamiana*
Q38922	14.21	110.58	$9.71E^{-12}$	AT4G35860.1, ATRABB1B, ATGB2, ATRAB2C, GB2,GTP-	ATGB2 (GTP-binding protein GB2) (Putative GTP-binding protein GB2). AT4g35860
P28188	37.76	259.44	$1.25E^{-23}$	AT1G02130.1, ATRAB1B, ARA5, ARA-5, ATRABD2A, RABD2A	Ras-related protein RABD2A (Ras-related protein ARA-5) (Ras-related protein RAB1B) (AtRab1b). RABD2A ARA-5 RAB1B
P92963	30.01	165.87	$5.82E^{-14}$	AT4G17170.1, AT-RAB2, ATRABB1C, ATRAB2A, RAB2A, RABB1C,	At4g17170 (GTP-binding RAB2A like protein) (Rab2-like protein). rab2 AT4g17170 dl4620c At4g17170
O80501	30.44	128.20	$2.87E^{-09}$	AT2G44610.1, RAB6, ATRABH1B, ATRAB6A, RAB6A	Ras-related protein RABH1B. RABH1B RAB6A At2g44610 F16B22.10
B9MUT7	198.68	384.23	$1.53E^{-07}$	AT1G02130.1, ATRAB1B, ARA5, ARA-5, ATRABD2A, RABD2A,	RAB GTPase = *Populus trichocarpa*

Enriched in AZ

P28186	310.18	231.48	$4.28E^{-04}$	AT3G46060.3, ARA3, RAB GTPase homolog 8A (RAB E)	Ras-related protein ARA-3. ARA-3 At3g46060 F12M12_30
D7TQ04	106.71	55.33	$9.65E^{-05}$	AT3G18820.1, ATRABG3F, ATRAB7B, RAB71, RABG3F, RAB7B	RAB GTPase = *Vitis vinifera*
B9HUI6	175.78	71.31	$6.47E^{-09}$	AT3G54840.1, ARA6, ATRABF1, ARA-6, ATRAB5C	RAB GTPase = *Populus trichocarpa*
Q01111	90.21	21.40	$8.40E^{-09}$	AT1G06400.1, ARA2, ATRABA1A, ATRAB11E	Ras-related protein YPT3 = *Nicotiana plumbaginifolia*
A5C9K9	15.50	0.00	$9.77E^{-04}$	AT2G43130.1, ARA4, ATRAB11F, ATRABA5C , ARA-4, RABA5C	RAB GTPase = *Vitis vinifera*
Q43463	40.45	0.00	$2.98E^{-08}$	AT4G09720.1, ATRABG3A, RABG3A	Ras-related protein Rab7 = Glycine max
O04486	46.08	0.00	$9.31E^{-10}$	AT1G09630.1, ATRAB11C, ATRABA2A , ATRAB-A2A, RAB-A2A,	Ras-related protein RABA2a (Ras-related protein Rab11C). RABA2A RAB11C At1g09630 F21M12.2
D7TIU1	50.55	0.00	$1.16E^{-10}$	AT5G03530.1, ATRAB ALPHA, ATRAB, ATRAB18B, ATRABC2A,	RAB GTPase = *Vitis vinifera*
A5AR55	62.98	0.00	$2.27E^{-13}$	AT1G07410.1, ATRABA2B, RAB-A2B, ATRAB-A2B, RABA2b	RAB GTPase = *Vitis vinifera*
A9PC79	117.11	0.00	$6.84E^{-49}$	AT2G44100.1, ATGDI1, AT-GDI1, GDI1, guanosine nucleotide	RAB GTPase = *Populus trichocarpa*

Small GTPase superfamily. ARF-like GTPase family

Enriched in fruit

P51824	0.00	79.52	$2.842171E^{-14}$	AT5G14670.1, ATARFA1B, ARFA1B, ADP-ribosylation factor	ADP-ribosylation factor 1 = *Solanum tuberosum*

Small GTPase superfamily. RAN family

Enriched in AZ

P54765	14.35	0.00	$1.95E^{-03}$	AT5G55190.1, RAN3, ATRAN3, RAN GTPase 3	GTP-binding nuclear protein Ran1 = *Lotus japonicus*

Small GTPase superfamily. Rho GTPase family

	Enriched in fruit					
	Q38912	0.00	106.06	$4.336809E^{-19}$	AT4G35020.3, RAC3	Rac-like GTP-binding protein ARAC3 (GTPase protein ROP6). ARAC3 RAC1 ROP6 At4g35020 M4E13.80
	Enriched in AZ					
	D7U1Z9	102.82	2.92	$1.515424E^{-59}$	AT5G27540.2, MIRO1	Mitochondrial Rho GTPase = *Vitis vinifera*
	Q6EP31	213.19	0.00	$7.34684E^{-40}$	AT1G75840.1, ARAC5, ATGP3,	Rac-like GTP-binding protein 5 (GTPase protein RacD) (OsRac5) = *Oryza sativa*

Small GTPase superfamily. SAR1 family

	Enriched in fruit					
	O04834	407.59	1017.27	$2.92844E^{-29}$	AT4G02080.1, ASAR1, ATSARA1C, ATSAR2, SAR	GTP-binding protein SAR1A. SAR1A At4g02080 T10M13.9 AGAA.4
**Dynamin family**						
	Enriched in fruit					
	B9MVC5	0.00	17.51	$2.33E^{-10}$	AT3G60190.1, ADL4, ADLP2, EDR3, DRP1E, ADL1E, DL1E	Dynamin = *Populus trichocarpa*
	Enriched in AZ					
	B9SRI2	11.32	0.00	$1.49E^{-08}$	AT3G19720.1, ARC5, DRP5B	Dynamin = *Ricinus communis*
	D7TGM4	10.14	0.00	$3.73E^{-09}$	AT1G59610.1, ADL3, CF1, DRP2B, DL3	Dynamin = *Vitis vinifera*
	D7SN01	12.88	0.00	$7.28E^{-12}$	AT1G10290.1, ADL6, DRP2A	Dynamin = *Vitis vinifera*
	D7U670	17.69	0.00	$8.88E^{-16}$	AT4G33650.1, ADL2, DRP3A	Dynamin = *Vitis vinifera*
	A5CA51	32.22	0.00	$2.17E^{-19}$	AT3G60190.1, ADL4, ADLP2, EDR3, DRP1E, ADL1E, DL1E	Dynamin = *Vitis vinifera*
**V-type ATPase family**						
	Enriched in fruit					
	D7SX66	0.00	25.13	$2.38E^{-07}$	AT3G28715.1, ATPase, V0/A0 complex, subunit C/D	V-type ATPase = *Vitis vinifera*
	Q9FLN5	28.57	147.61	$4.13E^{-05}$	AT5G55290.2, ATPase, V0 complex, subunit E	AT5G55290 protein (AT5g55290/MCO15_24) (Genomic DNA. chromosome 5. P1 clone:MCO15) AT5G55290 At5g55290
	P59227	516.26	1674.79	$3.12E^{-60}$	AT4G34720.1, VHA-C1 AVA-P1 AVAP1	V-type proton ATPase 16 kDa proteolipid subunit c1/c3/c5 (V-ATPase 16 kDa proteolipid subunit c1/c3/c5) (Vacuolar H(+)-ATPase
	Q9SZY7	57.40	120.37	$2.88E^{-03}$	At4g34720 T4L20.300; VHA-C3AVA-P1 , VHA-C1, ATVHA-C1 | ATPase,	H+-transporting ATPase-like protein (Vacuolar membrane ATPase subunit c'')
	D7T7L1	40.02	69.70	$4.16E^{-03}$	AT3G42050.1, vacuolar ATP synthase subunit H family protein	V-type ATPase = *Vitis vinifera*
	Enriched in AZ					
	D7SS06	252.00	190.47	$1.48E^{-07}$	AT1G78900.2, VHA-A, vacuolar ATP synthase subunit A	V-type ATPase = *Vitis vinifera*

	Q6KAA4	117.94	35.89	7.67E^{-06}	AT4G02620.1, vacuolar ATPase subunit F family protein	V-type proton ATPase subunit F = *Oryza sativa*
	Q9LJI5	494.77	50.33	6.20E^{-107}	AT3G28710.1, ATPase, V0/A0 complex, subunit C/D	V-type proton ATPase subunit d1 (V-ATPase subunit d1) (Vacuolar H(+)-ATPase subunit d. VHA-D1 At3g28710 MZN14.21
	Q9LX65	7.55	0.00	9.77E^{-04}	AT3G42050.1, vacuolar ATP synthase subunit H family protein	V-type proton ATPase subunit H (V-ATPase subunit H) (Vacuolar H(+)-ATPase subunit H) (Vacuolar proton pump subunit H). VHA-H
	B9S253	48.57	0.00	6.78E^{-21}	AT3G42050.1, vacuolar ATP synthase subunit H family protein	Vacuolar ATP synthase subunit h = *Ricinus communis*
	Q8RU33	242.16	0.00	6.75E^{-80}	AT3G28715.1, ATPase, V0/A0 complex, subunit C/D	Probable V-type proton ATPase subunit d (V-ATPase subunit d) (Vacuolar proton pump subunit d) = *Oryza sativa*
**Syntaxin/t-SNARE family**						
	Enriched in fruit					
	D7U8L0	0.00	13.02	4.88E^{-04}	AT5G08080.1, SYP132, ATSYP132 | syntaxin of plants 132	Syntaxin = *Vitis vinifera*
	B9SUS4	0.00	8.65	7.81E^{-03}	AT5G08080.1, SYP132, ATSYP132 | syntaxin of plants 132	Syntaxin = *Ricinus communis*
	Enriched in AZ					
	B9T2S1	243.53	108.06	7.08E^{-11}	AT5G06320.1, NHL3 | NDR1/HIN1-like 3	Syntaxin = *Ricinus communis*
	B9MU58	26.76	0.00	2.38E^{-07}	AT5G46860.1, VAM3, ATVAM3, SYP22, ATSYP22, SGR3 |	Syntaxin = *Populus trichocarpa*
	B9SDJ9	136.07	0.00	9.18E^{-41}	AT3G11820.1, SYP121, AT-SYR1, ATSYP121, SYR1, ATSYR1, PEN1 |	Syntaxin = *Ricinus communis*
**Others families**						
	B9S5U3	0.50	0.00	3.91E^{-03}		Midasin = *Ricinus communis*
	Q9SUT9	44.28	0.00	7.28E^{-12}	At4g11220 F8L21.10	Reticulon-like protein B2 (AtRTNLB2) (VirB2-interacting protein 2). RTNLB2 BTI2 At4g11220 F8L21.10

Table S4. Fruit- or AZ-enriched genes encoding various transport proteins at the last stage of olive fruit ripening. Sequences were selected after establishing a P<0.01. The table shows the total read count in RPKMx1000 for each gene after normalization across the two samples: fruit-pericarp and their AZ at 217 DPA.

	Uniprot ID	ZA	Fruit	P value	Description
Sugar transporter					
	D7SH34	0.00	696.00	1,50E-158	Sugar transporter= *Vitis vinifera*
	D7UDD8	0.00	185.00	1,10E-103	Major facilitator superfamily, Sugar transporter family = *Vitis vinifera*
	Q3E6T0	0.00	156.00	4,38E-46	Probable sugar phosphate/phosphate translocator At5g25400
	B9SJK9	0.00	102.00	1,06E-22	Sugar transporter = *Ricinus communis*
	D7U4Q9	0.00	56.00	6,94E-18	Sugar transporter = *Vitis vinifera*
	B9SBS6	0.00	43.00	5,68E-14	CMP-sialic acid transporter = *Ricinus communis*
	B9RM94	3.68	71.00	9,08E-13	Sugar transporter = *Ricinus communis*
	B9R8S3	51.87	470.00	4,59E-170	Sugar transporter = *Ricinus communis*
	Q68BJ8	285.36	386.00	5,01E-04	Sorbitol transporter = *Malus domestica*
	D7TBL1	17.08	2.00	1,52E-05	Monosaccharide transporter = *Vitis vinifera*
	B9RP33	6.55	0.00	7,81E-03	UDP-sugar transporter = *Ricinus communis*
	B9RTN4	4.52	0.00	7,81E-03	Sugar transporter = *Ricinus communis*
	Q9SX48	4.51	0.00	7,81E-03	Sugar transport protein 9 (Hexose transporter 9) STP9 At1g50310 F14I3.9
	D7TJV0	11.49	0.00	3,91E-03	Sugar transporter = *Vitis vinifera*
	Q9SBA7	6.57	0.00	9,77E-04	Sugar transport protein 8 (Hexose transporter 8) STP8 At5g26250 F9D12.9 T19G15.100
	D7T6L7	8.37	0.00	4,88E-04	ERD6-like transporter = *Vitis vinifera*
	Q94EI9	11.76	0.00	2,44E-04	Sugar phosphate/phosphate translocator At3g14410
	Q07423	9.8	0.00	3,05E-05	Hexose carrier protein HEX6 = *Ricinus communis*
	B9RTZ7	45.28	0.00	5,29E-23	Sugar transporter = *Ricinus communis*
	B9SZL0	85.85	0.00	1,84E-40	Sugar transporter = *Ricinus communis*
	B9RZB4	192.95	0.00	2,34E-97	Sugar transporter= *Ricinus communis*
N transporter					
	D7U9B7	0.00	115.00	2,69E-101	Oligopeptide transporter = *Vitis vinifera*
	D7TNF6	0.00	70.00	1,50E-36	Oligopeptide transporter = *Vitis vinifera*
	A5C8T7	0.00	44.00	5,29E-23	Oligopeptide transporter = *Vitis vinifera*
	D7U9C1	0.00	19.00	7,28E-12	Oligopeptide transporter = *Vitis vinifera*
	D7T4G2	0.00	12.00	9,54E-07	Oligopeptide transporter = *Vitis vinifera*
	D7SLW7	0.00	7.00	2,44E-04	Oligopeptide transporter = *Vitis vinifera*
	O80436	0.00	7.00	9,77E-04	Peptide/nitrate transporter At2g38100
	D7U9B1	1.39	69.00	3,33E-112	Oligopeptide transporter = *Vitis vinifera*
	B9SM54	6.45	23.00	1,45E-03	Amino acid transporter = *Ricinus communis*
	D7T9Q9	14.25	45.00	9,74E-08	Amino acid transporter = *Vitis vinifera*
	B9S4A3	23.65	61.00	3,30E-05	Amino acid transporter = *Ricinus communis*

D7TL72	17.23	9.00	9,40E-03	Amino acid transporter = *Vitis vinifera*
Q9ZTX4	42.53	4.00	8,45E-17	Oligopeptide transporter, LeOPT1 = *Solanum lycopersicum*
B9RBN4	103.03	8.00	9,43E-31	Amino acid transporter = *Ricinus communis*
D7TJT3	1.86	0.00	7,81E-03	Oligopeptide transporter = *Vitis vinifera*
B9RD13	4.69	0.00	3,91E-03	Cationic amino acid transporter = *Ricinus communis*
B9S4A2	6.06	0.00	3,91E-03	Amino acid transporter = *Ricinus communis*
B9T755	2.35	0.00	3,91E-03	Peptide transporter = *Ricinus communis*
Q9SRK7	5.76	0.00	9,77E-04	Adenine/guanine permease AZG1 (AzgA-homolog protein) (Protein AZAGUANINE RESISTANT 1) (AtAzg1), AZG1 At3g10960 F9F8.22
B9S1L4	7.54	0.00	4,88E-04	Amino acid transporter = *Ricinus communis*
D7T9A4	4.14	0.00	4,88E-04	Oligopeptide transporter = *Vitis vinifera*
Q9LFX9	6.94	0.00	2,44E-04	Nitrate transporter 1.6
B9RYS3	6.14	0.00	6,10E-05	Oligopeptide transporter = *Ricinus communis*
B9SI71	8.81	0.00	7,63E-06	Cationic amino acid transporter = *Ricinus communis*
B9SKU5	10.22	0.00	3,81E-06	Cationic amino acid transporter = Ricinus communis
Q9ZPR7	18.80	0.00	2,38E-07	Ureide permease 1 (AtUPS1), UPS1 At2g03590 F19B11.4
B9RPK4	11.43	0.00	1,19E-07	Oligopeptide transporter = *Ricinus communis*
A5BUN8	13.76	0.00	1,49E-08	Oligopeptide transporter = *Vitis vinifera*
B9RAN2	18.32	0.00	9,31E-10	Purine permease = *Ricinus communis*
B9SJX4	18.94	0.00	9,31E-10	GABA-specific permease = *Ricinus communis*
Q7XAK5	19.57	0.00	1,46E-11	Nitrate transporter = *Prunus persica*
B9R7I7	23.11	0.00	1,82E-12	Purine permease = *Ricinus communis*
B9T6M4	13.08	0.00	2,84E-14	Peptide transporter= *Ricinus communis*
B9S275	41.31	0.00	3,23E-27	Oligopeptide transporter = *Ricinus communis*
D7SZL3	54.05	0.00	1,58E-30	Oligopeptide transporter = *Vitis vinifera*
B9R934	162.79	0.00	6,14E-92	Nitrate transporter = *Ricinus communis*
D7SIH4	110.16	0.00	8,52E-109	Oligopeptide transporter = *Vitis vinifera*

Aquaporin

Q08733	0.00	61.00	8,88E-16	Aquaporin PIP1-3 (AtPIP1;3) (Plasma membrane intrinsic protein 1c) (PIP1c) (TMP-B), PIP1-3 PIP1C TMPB At1g01620 F22L4.16
B9RS20	0.00	38.00	7,45E-09	Tonoplast intrinsic protein, MIP/aquaporin family = *Ricinus communis*
C5IX25	16.61	225.00	2,30E-37	Plasma intrinsic protein 2;5, MIP/aquaporin family = Juglans regia
Q0MX13	26.28	319.00	3,33E-50	Aquaporin PIP2;2 = *Vitis vinifera*
A9P9G2	148.45	729.00	4,38E-61	Aquaporin, MIP family, TIP subfamily = *Populus trichocarpa*
C5IX20	27.87	84.00	1,18E-05	Plasma intrinsic protein 1;1, MIP/aquaporin family = *Juglans regia*
B5KGP0	361.00	181.00	1,07E-13	Small basic intrinsic protein 1-2, MIP/aquaporin family = *Olea europaea*
B9S0M0	84.49	36.00	5,05E-06	Aquaporin PIP2.2 = *Ricinus communis*

	A9PFE8	8.13	0.00	7,81E-03	Aquaporin, MIP family, PIP subfamily = *Populus trichocarpa*
	Q9AVB3	60.39	0.00	1,11E-16	Plasma membrane intrinsic protein 2-2, Py-PIP2-2 = *Pyrus communis*
	C8CE50	117.24	0.00	2,47E-32	Aquaporin, PIP1;1 = *Fragaria ananassa*
ABC transporter					
	B9RUV8	0.00	35.00	1,06E-22	ATP-dependent transporter, ABC transporter = *Ricinus communis*
	B9N856	125.69	203.00	3,07E-06	ABC transporter family protein = *Populus trichocarpa*
	B9R7N6	6.9	0.00	9,77E-04	Multidrug resistance pump = *Ricinus communis*
	B9RIN7	4.28	0.00	1,91E-06	Multidrug resistance-associated protein 1, 3 (Mrp1, 3), abc-transoprter = Ricinus communis
	B9R6R0	15.78	0.00	2,98E-08	Multidrug resistance pump = *Ricinus communis*
	B9S7S7	35.02	0.00	1,86E-09	Phosphate abc transporter = *Ricinus communis*
	B9T0A5	19.66	0.00	1,14E-13	ATP-binding cassette transporter = *Ricinus communis*
	D7UBX7	10.57	0.00	2,84E-14	ABC transporter = *Vitis vinifera*
	B9RXM4	67.97	0.00	6,16E-33	Multidrug resistance pump = *Ricinus communis*
	B9T6V3	71.92	0.00	6,16E-33	Multidrug resistance pump = *Ricinus communis*
	B9RXM3	71.09	0.00	1,54E-33	Multidrug resistance pump = *Ricinus communis*
	D7T9B7	122.29	0.00	1,73E-77	ABC transporter = *Vitis vinifera*
Metal transporter					
	A5C1P9	0.00	354.00	1,22E-92	Metal ion transporter= *Vitis vinifera*
	B9T4M0	0.00	111.00	9,18E-35	Urease accessory protein ureH = *Ricinus communis*
	B9RAC5	0.00	46.00	1,42E-14	Metal ion binding protein = *Ricinus communis*
	E0CUG6	0.00	136.00	1,14E-13	Metal ion transporter = *Vitis vinifera*
	D7TBR6	0.00	82.00	2,33E-10	Metal ion transporter = *Vitis vinifera*
	B9RFA4	0.00	10.00	9,77E-04	Chloroplast-targeted copper chaperone, Metal ion transporter = *Ricinus communis*
	D7SND2	0.00	9.00	7,81E-03	Metal ion transporter = *Vitis vinifera*
	A1YIQ6	16.19	150.00	5,93E-27	Zinc transporter = *Solanum lycopersicum*
	Q9FJH5	95.41	240.00	3,50E-11	At5g60800 (Gb|AAC98457.1) Metal ion transporter
	O82089	190.08	69.00	4,76E-07	AT3g56240/F18O21_200 (Copper homeostasis factor)
	D7TQQ5	184.54	57.00	3,91E-20	Metal ion transporter = *Vitis vinifera*
	A5C6S4	79.57	9.00	5,65E-08	Metal ion transporter = *Vitis vinifera*
	Q6R3K6	65.09	2.00	6,56E-37	Metal-nicotianamine transporter YSL6 (Protein YELLOW STRIPE LIKE 6) (AtYSL6), YSL6 At3g27020 MOJ10.9
	D7TV62	17.41	0.00	7,81E-03	Metal ion transporter = *Vitis vinifera*
	A5APE7	21.13	0.00	1,95E-03	Metal ion transporter = *Vitis vinifera*
	Q8RY06	6.91	0.00	1,95E-03	Metal ion transmembrane transporter At2g04305/T23O15.7
	D7T3V8	9.42	0.00	9,77E-04	Metal ion transporter = *Vitis vinifera*
	Q6R3L0	4.95	0.00	9,77E-04	Metal-nicotianamine transporter YSL1 (Protein YELLOW STRIPE LIKE 1) (AtYSL1), YSL1 At4g24120 T19F6.8 T19F6.110

	A5BBJ4	13.00	0.00	4,88E-04	Metal ion transporter = *Vitis vinifera*
	D7T8F6	7.59	0.00	4,88E-04	Metal ion transporter = *Vitis vinifera*
	D7UCD8	22.36	0.00	4,88E-04	Metal ion transporter = *Vitis vinifera*
	B9TAG3	189.39	0.00	6,62E-24	Metal ion binding protein = *Ricinus communis*
Nutrient transporter					
	B9SIK0	0.00	350.00	1,67E-99	2-oxoglutarate/malate translocator, chloroplast = *Ricinus communis*
	Q9FMF7	0.00	198.00	3,92E-94	2-oxoglutarate/malate translocator (At5g64290) (Putative 2-oxoglutarate/malate translocator) (Putative 2-oxoglutarate/malate translocator protein)
	B9RID8	0.00	89.00	3,30E-42	2-oxoglutarate/malate translocator, chloroplast = *Ricinus communis*
	B9RUG9	0.00	37.00	8,88E-16	Cation efflux protein/ zinc transporter = *Ricinus communis*
	B9SH00	0.00	8.00	9,54E-07	Cation-transporting atpase plant = *Ricinus communis*
	Q8GWP3	0.00	44.00	3,81E-06	Copper transporter 6 (AtCOPT6), COPT6 At2g26975
	B9SJ59	0.00	7.00	2,44E-04	Sulfate transporter = *Ricinus communis*
	B9RR93	0.00	28.00	4,88E-04	Copper transporter = *Ricinus communis*
	B9RH84	138.90	358.00	9,13E-151	Phosphatidylinositol transporter = *Ricinus communis*
	B9T1L0	3.45	30.00	4,52E-15	Cation-transporting atpase plant = *Ricinus communis*
	B9RWZ4	1.86	10.00	4,13E-05	Cation-transporting atpase plant = *Ricinus communis*
	B9RUY1	12.93	30.00	6,22E-03	Sulfate transporter = *Ricinus communis*
	B9RWT3	17.93	3.00	6,62E-05	Ammonium transporter = *Ricinus communis*
	B9RHZ9	56.74	7.00	2,12E-13	Thiosulfate sulfertansferase = *Ricinus communis*
	B9STR6	17.23	2.00	3,11E-08	Cyclic nucleotide-gated ion channel = *Ricinus communis*
	B9R709	18.20	2.00	5,91E-12	Cation-transporting atpase plant = *Ricinus communis*
	B9S7C7	3.18	0.00	7,81E-03	Potassium transporter = *Ricinus communis*
	B9SCE3	2.33	0.00	7,81E-03	Copper-transporting atpase p-type = *Ricinus communis*
	Q9SYG9	5.18	0.00	9,77E-04	Cation/calcium exchanger 4 (AtCCX4) (Protein CATION CALCIUM EXCHANGER 4), CCX4 At1g54115 F15I1.21
	B9RKV6	3.61	0.00	4,88E-04	Cation-transporting atpase plant = *Ricinus communis*
	A8D009	17.81	0.00	6,10E-05	Ferritin = *Ricinus communis*
	B9SZW3	14.30	0.00	1,19E-07	Inorganic phosphate transporter = *Ricinus communis*
	Q93YH1	13.18	0.00	4,77E-07	Sodium/hydrogen exchanger, NHX2 = *Solanum lycopersicum*
	B9SI63	26.89	0.00	1,78E-15	Sodium/potassium/calcium exchanger 6 = *Ricinus communis*
	B9RUZ6	24.88	0.00	4,44E-16	Boron transporter = *Ricinus communis*
	Q1JRA3	31.66	0.00	2,22E-16	Sodium/hydrogen exchanger, NHX3 = *Solanum lycopersicum*
	D0V1M5	34.08	0.00	1,39E-17	Sodium/hydrogen exchanger = *Vitis vinifera*
	Q9SV13	30.9	0.00	2,17E-19	Sulfate transporter 3.1 (AST12) (AtST1), SULTR3;1 ST1 At3g51895 F4F15.10 ATEM1.15
	B9RGU8	35.33	0.00	2,58E-26	Potassium transporter = *Ricinus communis*
	B9SF53	62.81	0.00	4,93E-32	Inorganic phosphate transporter = *Ricinus communis*

B9SPI0	71.43	0.00	3,85E-34	Nitrate transporter = *Ricinus communis*
B9RIV7	70.92	0.00	9,40E-38	Arsenite transport protein = *Ricinus communis*
Q1L4E2	158.84	0.00	1,15E-41	Ferritin = *Malus domestica*
Q9SFB0	91.25	0.00	2,80E-45	MATE efflux family protein FRD3 (MATE citrate transporter) (Protein DTX43) (Protein FERRIC REDUCTASE DEFECTIVE 3) (AtFRD3) (Protein MANGANESE ACCUMULATOR 1) [illegible] FRD3 DTX43 MAN1
B9SF95	614.34	0.00	7,75E-267	Nitrate transporter = *Ricinus communis*

Table S5. PCR-primers used in this study.

Primer	Sequence	Gene (Uniprot ID)
ERF5-F	5’-TTCATCCAGCATCGGAGTAAC-3’	ERF5 (Q9SXS8)
ERF5-R	5’-GAGGGCTGTGCATGCAAGCTC-3’	
ERF4-F	5’-GTTAATGGAGTTAAGGATAAG -3’	ERF4 (Q9LW49)
ERF4-R	5’-TCAAGCTCAACCGGCGGTTG-3’	
SnRK2.4-F	5’-TACCGGAGAGAAAACCCAACC-3’	SnRK2.4 (P43291)
SnRK2.4-R	5’-TATGCCTCCCCAACCAAAGCC-3’	
NPR1-F	5’-CTGAATAATGTGTCATATCCG-3’	NPR1 (B9H966)
NPR1-R	5’-ATGCTCATTCTTTGTTAGTTG-3’	
JAR1-F	5’-AACATCGACAAGAATACGGAG-3’	JAR1 (B0VXR3)
JAR1-R	5’-GTCTAAACGGTTGCAGCATTC-3’	
